# An EHR-based framework for modeling growth curves and constructing growth centile charts for genetic disorders

**DOI:** 10.1038/s41525-026-00592-x

**Published:** 2026-07-03

**Authors:** Cathy Shyr, Rory J. Tinker, Rebekah F. Brown, Adam Wright, Josh F. Peterson, John A. Phillips, S. Trent Rosenbloom, Lisa Bastarache

**Affiliations:** 1 Department of Biomedical Informatics, Vanderbilt University Medical Center, Nashville, TN USA; 2 Department of Pediatrics, Vanderbilt University Medical Center, Nashville, TN USA; 3 Department of Biostatistics, Vanderbilt University Medical Center, Nashville, TN USA; 4 Department of Medical Genetics and Genomics, Icahn School of Medicine at Mount Sinai, New York, NY USA; 5 Department of Medicine, Vanderbilt University Medical Center, Nashville, TN USA

**Keywords:** Computational biology and bioinformatics, Diseases, Genetics, Medical research

## Abstract

Growth modeling is central to human genetics, as deviations from typical growth can signal an underlying disorder. In this cohort study, we developed a generalizable framework for generating growth charts across genetic conditions using electronic health records (EHR). Leveraging 22 years of longitudinal EHR data from 452,470 patients across 15 genetic conditions and unaffected individuals, we generated sex- and condition-specific growth charts using Generalized Additive Models for Location, Scale, and Shape, and quantified differences in size, timing, and intensity using SuperImposition by Translation and Rotation (SITAR). SITAR-derived growth parameters showed strong concordance with established annotations in OMIM and Orphanet, and identified previously unreported growth patterns. We stratified cystic fibrosis by *CFTR* functional class and observed greater growth impairment in individuals with homozygous minimal-function variants compared to those with residual function. This framework provides a generalizable approach for leveraging EHR data to refine genotype-phenotype relationships and enable continuous updating of growth charts across genetic conditions.

## Introduction

Growth abnormalities are a common feature of many genetic disorders and serve as key indicators of underlying disease biology^1,2^. An understanding of growth abnormalities can help clinicians recognize genetic disorders in undiagnosed patients, interpret genetic test results more accurately, and monitor patient health more effectively after diagnosis^3–5^. While they are widely documented in knowledge bases such as Online Mendelian Inheritance in Man (OMIM)^6^ and Orphanet^7^, the growth abnormality descriptions in these resources are often categorical or qualitative (e.g., “tall stature,” “delayed growth”) and under-specified with respect to the actual growth magnitude, timing, and intensity. That is, they may indicate that a genetic disorder is associated with tall stature, but not quantify *how much* taller, *when* this deviation occurs, or the *rate* at which it occurs. Precise, longitudinal characterization is therefore needed to better define growth patterns in genetic disorders^8^.

Anthropometric data can be modeled in two complementary ways: growth curves, which describe age-related change in repeated growth measurements in individuals, and growth centiles, which describe the distribution of measurements in a population as a function of age^9^. Widely used references such as those from the Centers for Disease Control and Prevention (CDC)^10^ and World Health Organization^11^ are growth centile charts, representing population-level growth patterns in the general population. However, these references do not adequately capture the distinct growth patterns associated with many genetic disorders; moreover, they do not directly quantify clinically meaningful features such as size, timing, and intensity. To address this gap, condition-specific growth references have been developed for a small number of disorders, including Trisomy 21^12,13^, Prader-Willi syndrome^14^, and Turner syndrome^15,16^, but many genetic disorders still lack dedicated growth charts^17^. Moreover, existing condition-specific growth curves and centile charts have limitations. Many were derived from historical cohorts created before advances in diagnosis and treatment reshaped the natural history of these disorders^18–22^. Others primarily reflect data from individuals with severe or classic disease presentations, failing to capture the broader clinical spectrum now recognized through widespread adoption of next-generation sequencing^23^. These limitations underscore the need for scalable approaches that leverage real-world data to generate updated, condition-specific growth references across a broad range of genetic disorders.

In this study, we present a generalizable electronic health record (EHR)-based framework for modeling height in genetic conditions using two complementary approaches: Generalized Additive Models for Location, Scale, and Shape (GAMLSS)^24^ to estimate population growth centiles, and SuperImposition by Translation and Rotation (SITAR)^25^ to model individual growth curves and compare differences in size, timing, and intensity between affected and unaffected cohorts^24^. This framework consists of six analytic steps designed to be broadly applicable across genetic conditions and adaptable to different EHR systems. Leveraging 22 years of longitudinal EHR data from individuals with genetically confirmed diagnoses at a single academic medical center, we generated height growth curves and centile charts for 15 genetic disorders (Table 1). By systematically comparing affected and unaffected cohorts, we recapitulated known growth phenotypes in OMIM and Orphanet and identified previously under-recognized features. Furthermore, when comparing classic to non-classic cystic fibrosis, we found that our framework was sensitive to genotype-specific variation. We published an open-access resource featuring these condition-specific growth centile charts to enable broader use. Growth charts generated by this framework can be iteratively updated as EHR data accrue, providing a scalable foundation for refining growth phenotypes and enabling more context-specific and personalized growth modeling across genetic disorders.

**Table 1:** Overall summary of study cohort for growth modeling

	Number of individuals			Number of measurements Median (IQR)		
	Total	Male	Female	Total	Male	Female
**Total**	452,470	224,316	228,154	4 (3–5)	4 (3–5)	4 (3–5)
**Stratified by Condition**						
Unaffected	449,513	222,712	226,801	4 (3–5)	4 (3–5)	4 (3–5)
Charcot-Marie-Tooth disease, type 1 A	95	45	50	5 (3–10)	6 (3–9)	5 (3–10)
Cystic fibrosis	553	291	262	28 (8–61)	27 (7–59)	29.5 (9–63.8)
DiGeorge syndrome	227	103	124	12 (5–25)	14 (5–23.5)	12 (3.8–26)
Down syndrome	871	486	385	10 (4–20)	10 (4–23)	10 (4–19)
Duchenne muscular dystrophy	164	164	N/A	6 (3–12)	6 (3–12)	N/A
Fragile X Syndrome	83	62	21	4 (2–8)	4 (2–8)	5 (3–9)
Hemophilia A	83	83	N/A	14 (6.5–22.5)	14 (6.5–22.5)	N/A
Hemophilia B	33	33	N/A	12 (6–18)	12 (6–18)	N/A
Klinefelter syndrome	118	118	N/A	8 (3–15)	8 (3–15)	N/A
Marfan syndrome	99	44	55	10 (4–18.5)	11 (4.8–17)	10 (4–19)
Myotonic dystrophy 1	41	21	20	10 (5–16)	9 (6–15)	11.5 (5–16.5)
Neurofibromatosis, type 1	207	108	99	7 (3–14)	7 (3–14)	8 (3–14)
Prader-Willi syndrome	67	28	39	16 (4–40.5)	16.5 (5–39.5)	16 (4–40.5)
Turner syndrome	274	N/A	274	17 (8–31)	N/A	17 (8–31)
Williams-Beuren syndrome	46	21	25	13.5 (5–30.8)	12 (5–24)	25 (6–31)

N/A indicates not applicable, where the condition is not biologically possible in that subgroup (e.g., sex-specific conditions).

*IQR* interquartile range.

## Results

This cohort study included 452,470 pediatric patients (49.6% male, 50.4% female) with at least three encounters at VUMC between January 1, 2002, and December 31, 2023 (Table 1). A total of 2,253,058 height measurements were recorded between the ages of 2 and 20. The median number of measurements was 4 (interquartile range, 3–5) per patient.

### EHR-based growth modeling captures genetic condition-specific phenotypic signatures

**Fig. 1:**
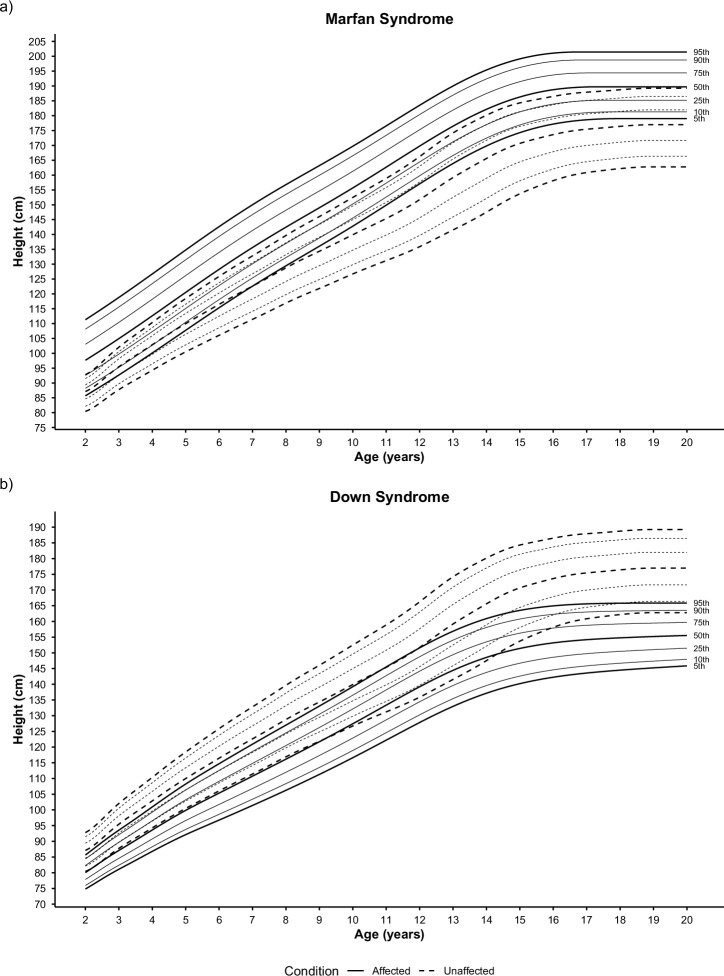
Height centile charts for boys with Marfan syndrome and Down syndrome. GAMLSS-fitted height centile charts for boys with **a** Marfan syndrome and **b** Down syndrome. Dashed lines represent affected individuals, and solid lines unaffected individuals.

**Fig. 2:**
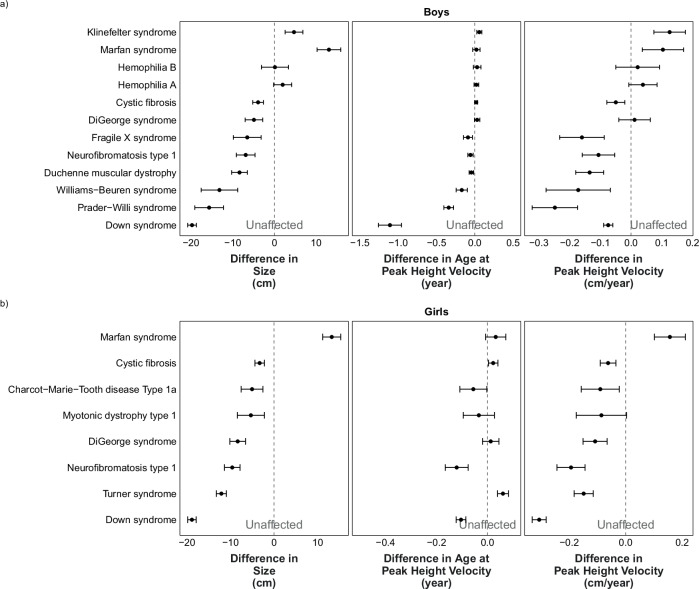
Sex-specific comparisons of growth parameters between individuals affected by genetic conditions and the unaffected cohort. Growth parameter comparisons are shown for **a** boys and **b** girls. Each bar represents the 95% confidence interval (CI) of the difference in maximum height, age at peak height intensity, or peak height intensity relative to the unaffected cohort (vertical dashed line).

All sex-specific, height-for-age growth centile charts from age 2 to 20 for the 15 genetic conditions and unaffected individuals are publicly available through our interactive RShiny application (https://cathyshyr.shinyapps.io/GrowthCharts/). We generated condition- and sex-specific growth charts for Charcot-Marie-Tooth disease type 1 A, cystic fibrosis, DiGeorge syndrome, Down syndrome, Duchenne muscular dystrophy, Fragile X syndrome, hemophilia A, hemophilia B, Klinefelter syndrome, Marfan syndrome, myotonic dystrophy type 1, neurofibromatosis type 1, Prader-Willi syndrome, Turner syndrome, and Williams-Beuren syndrome (Table 1). Given the large number of genetic conditions covered, we provide all growth centile charts in the Supplementary Information. Figure 1 shows the GAMLSS-fitted height centile charts for boys with Marfan syndrome (top) and Down syndrome (bottom) as representative examples. These centile charts demonstrate clear, condition-specific deviations in growth patterns from the unaffected cohort across age. For Marfan syndrome, height centiles are shifted upward relative to the unaffected cohort, consistent with increased stature. In contrast, Down syndrome shows a downward shift, reflecting reduced stature throughout childhood and adolescence. SITAR-derived coefficient estimates further quantified these differences across size, timing (age at peak height velocity), and intensity (peak height velocity) (Fig. 2). Individuals with Marfan syndrome were significantly taller than unaffected peers (boys: 13.1 cm taller (95% CI: 10.2, 15.9); girls: 13.2 cm taller (95% CI: 11.2, 15.3)), with taller stature also observed in Klinefelter syndrome and hemophilia among boys. In contrast, reduced height was observed across multiple conditions, including syndromic or chromosomal disorders (e.g., Down syndrome, Turner syndrome, Williams-Beuren syndrome) and systemic conditions (e.g., cystic fibrosis). Growth timing also varied across genetic conditions. Boys with Down syndrome reached peak height velocity 1.1 years earlier than unaffected peers (95% CI: 1, 1.3); similarly, individuals with Williams-Beuren syndrome, neurofibromatosis type 1, and Prader-Willi syndrome also reached peak height velocity earlier than the unaffected cohort. Peak height velocity was reduced in several conditions, including Prader-Willi syndrome, cystic fibrosis, DiGeorge syndrome, Turner syndrome, and neurofibromatosis type 1, whereas increased growth intensity was observed in Marfan syndrome and Klinefelter syndrome (Fig. 2).

### Genotype-specific growth patterns in cystic fibrosis

**Fig. 3:**
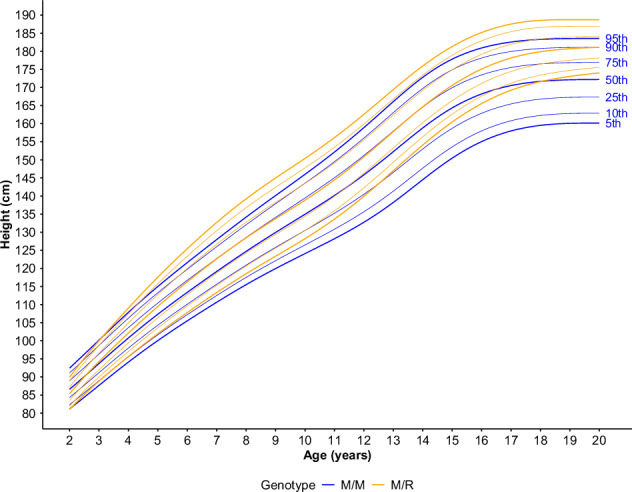
Height-for-age growth centile chart for boys with cystic fibrosis stratified by *CFTR* genotype. M = minimal function; R = residual function. Orange represents individuals with the M/R genotype, and blue the M/M genotype.

To assess intra-disorder heterogeneity, we stratified cystic fibrosis by *CFTR* functional class. A single SITAR model was fitted for individuals with homozygous minimal function (M/M) *CFTR* variants and those with minimal/residual function (M/R) genotypes^26^, with genotype status (M/M vs. M/R) included as a fixed effect for size, timing, and intensity. Among boys, M/M genotypes were associated with significantly reduced growth compared to M/R genotypes, including shorter maximum height (−5.2 cm (95% CI: −9.7, −0.8)) and lower growth intensity (−1.1 cm/year (95% CI: −2.7, −0.1)) (Fig. 3). No significant differences in size, timing, or intensity were observed among girls (Supplementary Information).

### Validation against growth phenotype annotations in OMIM and orphanet

To evaluate concordance with existing knowledge bases, we compared SITAR-derived growth parameter estimates with growth-related HPO terms in OMIM and Orphanet (Supplementary Table 1). Combining HPO terms from both sources revealed inconsistencies in terminology and coverage. These included differences in granularity (e.g., “tall stature” versus “disproportionate tall stature” for Marfan syndrome), incomplete annotation of known features (e.g., delayed puberty for Down syndrome present only in Orphanet), and, in some cases, conflicting annotations (e.g., both “short stature” and “tall stature” in neurofibromatosis type 1; Supplementary Table 2). Overall, SITAR-derived coefficient estimates showed high concordance with known growth abnormalities, correctly identifying 16 of 18 annotation-positive controls (sensitivity = 0.89) (Table 2). Among annotation-negative comparisons, 18 showed no significant difference, and 22 revealed new growth patterns not represented in OMIM or Orphanet (Table 2).

**Table 2:** Comparison of SITAR models’ coefficient estimates between individuals affected with genetic conditions and the unaffected cohort across size (taller vs. shorter), timing (earlier vs. later), and intensity (higher vs. lower). Superscripts indicate whether results are concordant with, discordant with, or not documented by Human Phenotype Ontology (HPO) terms in the Online Mendelian Inheritance in Man and Orphanet knowledgebases

		SITAR-derived coefficient estimates					
		Male			Female		
Condition	HPO terms from OMIM and Orphanet	Size	Timing	Intensity	Size	Timing	Intensity
Marfan syndrome	Size: Disproportionate tall stature, Tall stature	Taller^a^	No sig. diff.^a^	Higher^b^	Taller^a^	No sig. diff.^a^	Higher^b^
Neurofibromatosis, type 1	Size: Short stature, Overgrowth, Tall statureTiming: Delayed puberty, Precocious puberty	Shorter^a^	Earlier^a^	No sig. diff.^a^	Shorter^a^	Earlier^a^	No sig. diff.^a^
Prader-Willi syndrome	Size: Short stature Timing: Delayed puberty, Precocious puberty, Premature adrenarche Intensity: Growth delay	Shorter^a^	Earlier^a^	Lower^a^	Not Evaluable		
DiGeorge syndrome	Size: Short stature	Shorter^a^	No sig. diff.^a^	No sig. diff.^a^	Shorter^a^	No sig. diff.^a^	Lower^b^
Down syndrome	Size: Short stature Timing: Delayed puberty	Shorter^a^	Earlier^c^	Lower^b^	Shorter^a^	Earlier^c^	Lower^b^
Williams-Beuren syndrome	Size: Short stature Timing: Early onset of sexual maturation, Precocious puberty	Shorter^a^	Earlier^a^	Lower^b^	Not Evaluable		
Cystic fibrosis		Shorter^b^	No sig. diff.^a^	Lower^b^	Shorter^b^	No sig. diff.^a^	Lower^b^
Turner syndrome	Size: Short stature Timing: Delayed puberty Intensity: Growth delay, Postnatal growth retardation	Not Applicable			Shorter^a^	Later^a^	Lower^a^
Duchenne muscular dystrophy		Shorter^b^	No sig. diff.^a^	Lower^b^	Not Applicable		
Hemophilia B		No sig. diff.^a^	No sig. diff.^a^	No sig. diff.^a^	Not Applicable		
Hemophilia A		No sig. diff.^a^	No sig. diff.^a^	No sig. diff.^a^	Not Applicable		
Fragile X Syndrome		Shorter^b^	Earlier^b^	Lower^b^	Not Evaluable		
Myotonic dystrophy 1		Not Evaluable			Shorter^b^	No sig. diff.^a^	No sig. diff.^a^
Charcot-Marie-Tooth disease, type 1 A		Not Evaluable			Shorter^b^	Earlier^b^	Lower^b^
Kleinfelter syndrome		Taller^b^	Later^b^	Higher^b^	Not Applicable		

“No sig. diff.” indicates that the SITAR model’s coefficient estimate comparing affected and unaffected cohorts across size, timing, or velocity was not statistically significant. “Not evaluable” indicates that reliable coefficient estimate could not be obtained (e.g., due to model non-convergence or insufficient data to support a stable fit). “Not applicable” indicates that a condition is not biologically feasible for a specific sex (e.g., Turner syndrome in males).

*EHR* electronic health records.

^a^Concordant with knowledge bases.

^b^New growth pattern not documented in knowledge bases.

^c^Discordant with knowledge bases.

## Discussion

In this study, we present a generalizable EHR-based framework for modeling growth across genetic conditions, using GAMLSS to estimate population-level growth centiles and SITAR to model growth curves and quantify differences in size, timing, and intensity. Applied to 22 years of longitudinal data from over 452,470 individuals, this approach enabled systematic characterization of growth patterns across 15 genetic disorders. We demonstrated that EHR-derived growth modeling can both recapitulate established growth phenotypes and reveal additional dimensions of growth variation, including genotype-specific variation in cystic fibrosis and differences in growth timing and intensity that are not routinely captured in existing knowledge bases. Importantly, the growth charts derived from this study should be viewed as dynamic and iterative rather than definitive; as additional EHR data accrue, they can be continuously updated, refined, and extended toward more personalized representations of growth by incorporating factors such as ancestry, treatment exposures, and other clinically relevant covariates.

Validating our results against existing knowledge bases and published growth references revealed both concordance with established phenotypes and important differences, underscoring the potential of EHR-based growth modeling to refine and expand the phenotypic spectrum of genetic disorders. In conditions with established growth charts (e.g., Down syndrome^13^, cystic fibrosis^27^, Marfan syndrome^28–30^), our EHR-derived curves showed broad agreement, with minor deviations likely reflecting differences in study population, sample size, and evolving standards of care. Some observed differences, though absent from existing knowledge bases, are well supported by the literature. For example, the impact of Duchenne muscular dystrophy and cystic fibrosis on height and growth intensity is well documented^31^. Studies on Down syndrome have described both earlier age at peak height velocity and reduced peak height velocity^32,33^. Similarly, abnormalities in pubertal growth and stature have been reported in Fragile X syndrome, aligning with the reduced growth intensity and shorter stature observed in this study^34^. In Klinefelter syndrome, the combination of taller stature and increased growth intensity is consistent with known prior findings^35^. Other findings such as shorter stature, earlier pubertal timing, and reduced growth velocity in females with Charcot-Marie-Tooth disease type 1 A are not well characterized in the literature and should be considered exploratory observations requiring validation in independent cohorts.

Importantly, our framework was shown to be sensitive to genotype-specific variation within a given disorder. Stratification of individuals with cystic fibrosis by *CFTR* functional class revealed genotype-specific differences in both stature and growth dynamics, consistent with the more severe systemic impact of minimal function *CFTR* variants^26^. The cystic fibrosis analysis also illustrates how EHR-based growth modeling can identify age windows during which subgroup-specific growth phenotypes emerge, a concept consistent with prior longitudinal genetic association studies emphasizing that genetic effects on quantitative traits may vary across age^36^. Among boys, M/M and M/R genotype groups showed relatively similar height trajectories earlier in childhood, followed by clearer divergence during adolescence at higher centiles. Together with the SITAR-based finding of reduced growth intensity in boys with M/M genotypes, this pattern suggests that *CFTR* functional class may influence adolescent growth patterns. More broadly, these results suggest that EHR-based growth modeling may extend beyond disorder-level characterization to capture subgroup-specific variation (e.g., stratifying by treatment status or additional clinical variables), to generate new insights into the biological mechanisms underlying growth abnormalities. Because our EHR-derived growth curves were generated using data collected over 22 years of clinical practice, they reflect the combined influence of underlying disease biology, evolving standards of care, and advancements in diagnostic practice. This provides clinically relevant insight into real-world growth patterns, while also highlighting opportunities for future work to stratify by treatment exposure to distinguish natural history from management-related influences.

This study has several limitations. Our data were from a single academic center, which may limit generalizability. Further work is needed to evaluate how the growth curves and centile charts derived from this study perform across diverse populations or under other factors known to influence growth^37^. Another potential limitation is that SITAR assumes the same underlying growth curve for all individuals after adjusting for differences in size, timing, and intensity. As such, it may not be well suited to capture highly irregular or non-monotonic growth patterns that result from episodic illness or other disruptions to typical growth trajectories. In addition, sensitivity to alternative data inclusion criteria and secular trends over time could be explored in future work. Finally, while this study establishes a framework for modeling height trajectories, future work includes extending this framework to weight and head circumference data to support more comprehensive growth assessments. This requires addressing distinct challenges associated with these data in EHR, including secular trends in weight and data sparsity for head circumference beyond infancy.

This work establishes an EHR-based framework for growth modeling in genetic disorders. By refining known phenotypes, identifying under-recognized features, and detecting genotype-specific differences, this approach advances characterization of genotype-phenotype relationships. Broader application of EHR-based growth modeling has the potential to enrich existing knowledge bases and systematically expand our understanding of growth variation across genetic disorders.

## Methods

**Fig. 4:**
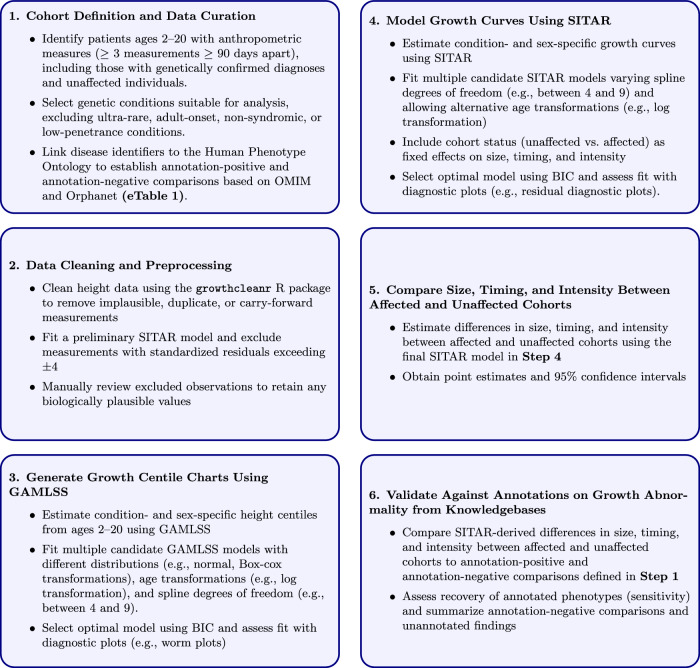
Overview of proposed EHR-based framework for modeling growth curves and growth centiles. EHR electronic health records, GAMLSS Generalized Additive Models for Location, Scale, and Shape; growthcleanr R package, SITAR SuperImposition by Translation and Rotation, BIC Bayesian Information Criterion, OMIM Online Mendelian Inheritance in Man knowledgebase.

Figure 4 summarizes our EHR-based framework for modeling growth curves and centiles, comprising six analytic steps designed to be broadly applicable across genetic conditions and adaptable to different EHR systems.

### Step 1. Cohort definition and data curation

This cohort study was based on patients seen at Vanderbilt University Medical Center (VUMC) between January 1, 2002, and December 31, 2023. Our cohort included patients between the ages of 2 and 20 with a genetically confirmed diagnosis and a minimum of three encounters at least 90 days apart. This study was approved by the Vanderbilt University Medical Center Institutional Review Board (IRB #171011) as exempt research. All procedures were performed in accordance with the ethical standards of the institutional research committee and with the Declaration of Helsinki. The requirement for informed consent was waived by the Institutional Review Board due to the retrospective nature of the study and secondary use of de-identified data. This study followed the Strengthening the Reporting of Observational Studies in Epidemiology (STROBE) reporting guidelines.

Our study used patient data derived from the Research Derivative, an image of VUMC’s EHR^38^. Within this dataset, patients with genetic disorders were identified in the clinical genetic database, a comprehensive database of clinical genetic testing data results developed at VUMC^39^, with genetically confirmed diagnoses annotated with identifiers from OMIM^6^ and/or Orphanet^7^. To define the set of disorders for analysis, we excluded diagnoses that were too rare (i.e., defined as having fewer than 10 male or 10 female cases), cancer-predisposition syndromes primarily associated with adult malignancy risk, genetic risk factors with low penetrance that do not produce characteristic phenotypes, non-syndromic conditions that affect only a single organ system, and late-onset disorders that typically do not manifest until adulthood. After applying these criteria, we identified 15 genetic disorders for sex-specific growth modeling (Table 1).

To compile a list of known growth abnormalities associated with genetic disorders, we used clinical descriptions from OMIM and Orphanet. These descriptions have been mapped to the Human Phenotype Ontology (HPO), a standardized vocabulary designed to describe phenotypes observed in rare and genetic disorders^40^. We identified all descendants of the HPO terms for disorders of growth (HP:0001507) and puberty (HP:0100000) and linked them to disease IDs in OMIM and Orphanet. To assess the overall relevance of growth abnormalities in rare and genetic disorders, we analyzed the proportion of OMIM and Orphanet diseases that included these terms.

We categorized height-related HPO terms into three domains: size (shorter versus taller), timing (earlier versus later), and intensity (lower versus higher). For example, “Short stature” (HP:0004322) was classified as shorter size, “Delayed puberty” (HP:0000823) as later timing, and “Postnatal growth retardation” (HP:0008897) as lower intensity. When disease descriptions were available in both OMIM and Orphanet, we merged the HPO terms from the two knowledgebases and analyzed their overlap.

### Step 2. Data cleaning and preprocessing

Demographic information, including sex and date of birth, was retrieved from the person table in the Observational Medical Outcomes Partnership (OMOP) common data model, which standardizes EHRs for research use. Height measurements ascertained between 2002/01/01 and 2024/03/31 were retrieved from the measurement table in OMOP. We used growthcleanr^41^, a validated data cleaner for anthropometric measurements in pediatric EHR data, to remove implausible height measurements. Briefly, these included measurements with extreme values, carried-forward entries, or duplicates. We further applied a model-based cleaning procedure using SITAR^25^, a nonlinear mixed effects model for longitudinal growth data, and excluded observations with standardized residuals exceeding ±4^9^. Because some monogenic conditions are associated with extreme but valid growth patterns, we manually reviewed excluded measurements and identified three patients whose data were removed due to extreme height deviations: Marfan syndrome (*n* = 2), and Prader-Willi syndrome (*n* = 1). These individuals were added back into the final dataset for analysis.

### Step 3. Generate growth centile charts using GAMLSS

We estimated sex-specific, condition-specific height centiles from ages 2 to 20 using Generalized Additive Models for Location, Scale, and Shape (GAMLSS)^24^, the standard framework for constructing growth centiles and an extension of the LMS method^42^. Let $Y$ denote height$.$ Height measurements were treated as independent cross-sectional observations. To mitigate overrepresentation of individuals with dense longitudinal follow-up, we retained one observation per individual within each six-month age interval prior to GAMLSS fitting. We assumed that $Y\sim {f}_{Y}\left(y| \mu ,\sigma ,\nu ,\tau \right)$, where ${f}_{Y}$ is a parametric distribution, and $\mu ,\sigma ,\nu ,$ and $\tau$ the parameters for location, scale, skewness, and kurtosis, respectively. We modeled each parameter as a smooth function of age using penalized B-splines. We fitted multiple candidate GAMLSS models, including four distributions commonly used in growth modeling (normal, Box-Cox Cole and Green^42^, Box-Cox power exponential, and Box-Cox $t$) and alterative age transformations (log or square root). The smoothness of the age functions was tuned by cross-validation, and the final optimal model was selected by minimizing the Bayesian Information Criterion (BIC)^43^. Model fit was assessed using worm plots and quantile-quantile plots of residuals. As an additional descriptive check of fit and clinical plausibility, we visually compared the fitted centiles (5th, 10th, 25th, 50th, 75th, 90th, and 95th percentiles) with the observed height data across age. Analyses were performed using the gamlss package in R (v.4.4.2)^44,45^.

### Step 4. Model growth curves using SITAR

We estimated sex-specific, condition-specific height curves from ages 2 to 20 using the SuperImposition by Translation And Rotation (SITAR)^25^ model, a nonlinear mixed effects model for longitudinal growth data. SITAR models the mean growth trajectory as a natural cubic regression B-spline, in addition to a set of population-level characteristics (fixed effects) and patient-specific effects (random effects). These effects correspond to three clinically interpretable aspects of growth: size, timing, and intensity^10,11^. For each genetic condition, we fitted SITAR models separately for boys and girls using data from both affected and unaffected individuals. To compare affected and unaffected cohorts, we included cohort status as fixed effects on the size, timing, and intensity parameters, such that the corresponding coefficients directly estimate the mean differences between affected and unaffected cohorts. The SITAR model is shown in Eq. (1):

1 $${y}_{{ij}}={a}_{0}+{a}_{{\rm{Affected}}}+{{\rm{\alpha }}}_{i}+H\left(\frac{g\left({t}_{j}\right)-{b}_{0}-{b}_{{\rm{Affected}}}-{{\rm{\beta }}}_{i}}{\exp \left(-{c}_{0}-{c}_{{\rm{Affected}}}-{{\rm{\gamma }}}_{i}\right)}\right)+{{\rm{\epsilon }}}_{{ij}},$$

where ${y}_{{ij}}$ denotes the height measurement of individual $i$ at age ${t}_{j}.$ The fixed effects for size, timing, and intensity are denoted by ${a}_{0},{b}_{0},$ and ${c}_{0}$, respectively, the random effects by ${{\rm{\alpha }}}_{i},{{\rm{\beta }}}_{i},{{\rm{\gamma }}}_{i}$ and the cohort status fixed effects by ${a}_{{\rm{Affected}}},{b}_{{\rm{Affected}}},$ ${c}_{{\rm{Affected}}}.$ The function $g\left(\cdot \right)$ is the age transformation, and $H\left(\cdot \right)$ is the mean growth trajectory modeled using a natural cubic B-spline. Residual errors ${{\rm{\epsilon }}}_{{ij}}$ were assumed to be normally distributed, and the random effects were assumed to follow a multivariate normal distribution. We fitted multiple candidate SITAR models varying the spline degrees of freedom from four to nine and allowing alternative age transformations (log or square root). The final optimal model was selected by minimizing BIC^43^. Model fit was assessed using residual diagnostic plots and quantile-quantile plots of residuals and random effects. Analyses were performed using the sitar and nlme packages in R (v.4.4.2)^44^.

### Step 5. Compare size, timing, and intensity between affected and unaffected cohorts

We quantified differences in growth between affected and unaffected individuals using the SITAR fixed-effect estimates for cohort status. For each genetic condition and sex, these coefficients represent the mean differences in size, timing (age at peak height velocity), and intensity (peak height velocity) between cohorts. Point estimates and corresponding 95% Wald confidence intervals (CI) were obtained from the fitted models^35^.

### Step 6. Validate against annotations on growth abnormality from knowledge bases

We compared SITAR-derived differences in size, timing, and intensity between affected and unaffected individuals to growth abnormality annotations in OMIM and Orphanet. The unit of analysis was the condition–sex–domain combination, where domain corresponded to size, timing, or intensity. We defined annotation-positive controls as condition-sex-domain combinations for which a growth abnormality has already been documented in OMIM or Orphanet. These serve as “known positives” that the SITAR-fitted models should ideally recover. On the other hand, an annotation-negative comparison refers to a condition-sex-domain combination for which no growth abnormality has been documented in these resources. To illustrate with an example, for Marfan syndrome, OMIM and Orphanet have the annotation “tall stature,” which corresponds to an abnormality in size. Therefore, *Marfan syndrome-male-size* is treated as an annotation-positive control, and we assess whether the SITAR model’s coefficient estimate for cohort status (unaffected vs. affected) for size is directionally correct and statistically significant (i.e., concordant with OMIM/Orphanet). Conversely, *Marfan syndrome-male-timing* has no existing annotations in OMIM or Orphanet and is therefore treated as an annotation-negative comparison. If the SITAR model’s timing parameter estimate for the difference between affected vs. unaffected cohorts are not statistically significant, we classify this as concordant with OMIM/Orphanet; otherwise, we classify this as a new growth pattern not represented in OMIM or Orphanet. All statistical analyses were performed using R (v.4.4.2)^44^. The annotated- positive and negative datasets are provided in the Supplementary Information.

## Supplementary information

Supplement_Final_R1

## Data Availability

Data used in this study were derived from the electronic health record (EHR) system at Vanderbilt University Medical Center. These data contain protected health information and are subject to institutional and legal restrictions that prevent public sharing.

## Code availability

Code used to generate and compare growth curves in this study can be accessed at the public Github repository: https://github.com/cathyshyr/growth_charts. We provide all growth curves on our open access RShiny application: https://cathyshyr.shinyapps.io/GrowthCharts/.
