# Supplementary material for: An EHR-based framework for modeling growth curves and constructing growth centile charts for genetic disorders
Source: NPJ Genom Med. 2026 Jul 3;11:39. doi: 10.1038/s41525-026-00592-x (PMC13338247; doi:10.1038/s41525-026-00592-x)
Supplement: Supplementary file 1 — Supplement_Final_R1 [file 41525_2026_592_MOESM1_ESM.pdf]

Supplementary Information

Supplementary Figure 1. Condition: Charcot Marie Tooth Disease Type 1a, Sex: Female

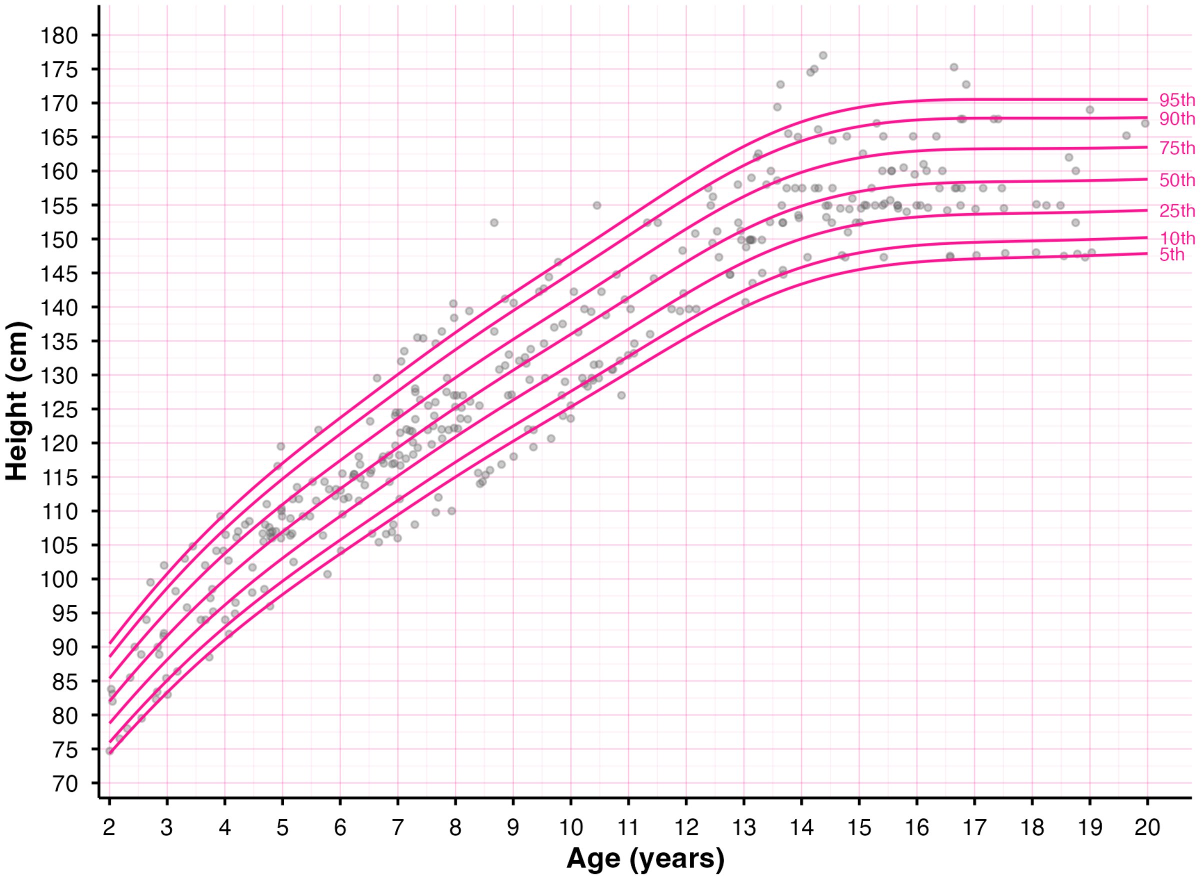

age_bin	N Observations
2-4	36
4-6	49
6-8	76
8-10	47
10-12	33
12-14	44
14-16	46
16-18	26
18-20	14

Supplementary Figure 2. Condition: Cystic Fibrosis, Sex: Female, Genotype: M/M

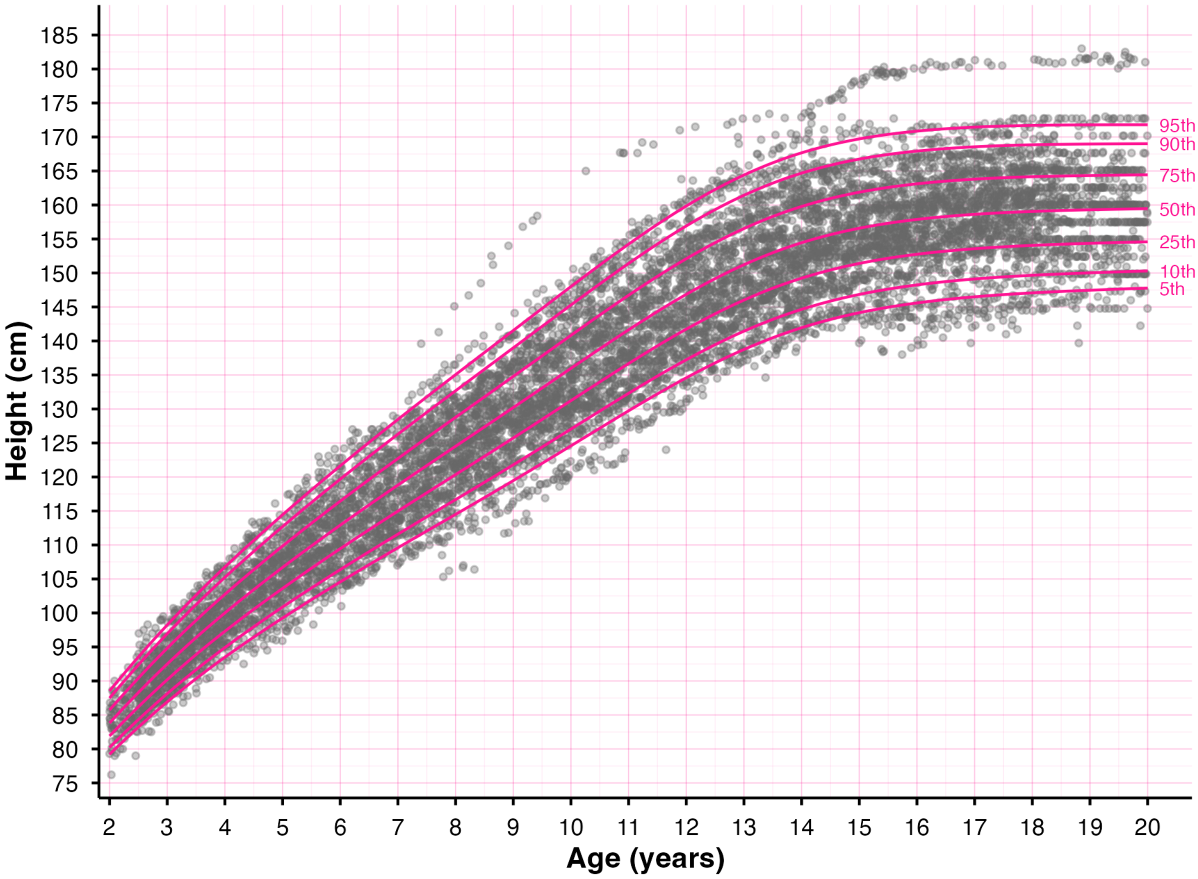

age_bin	N Observations
2-4	855
4-6	949
6-8	1,032
8-10	1,171
10-12	1,156
12-14	1,190
14-16	1,216
16-18	1,191
18-20	761

Supplementary Figure 3. Condition: Cystic Fibrosis, Sex: Female, Genotype: M/R

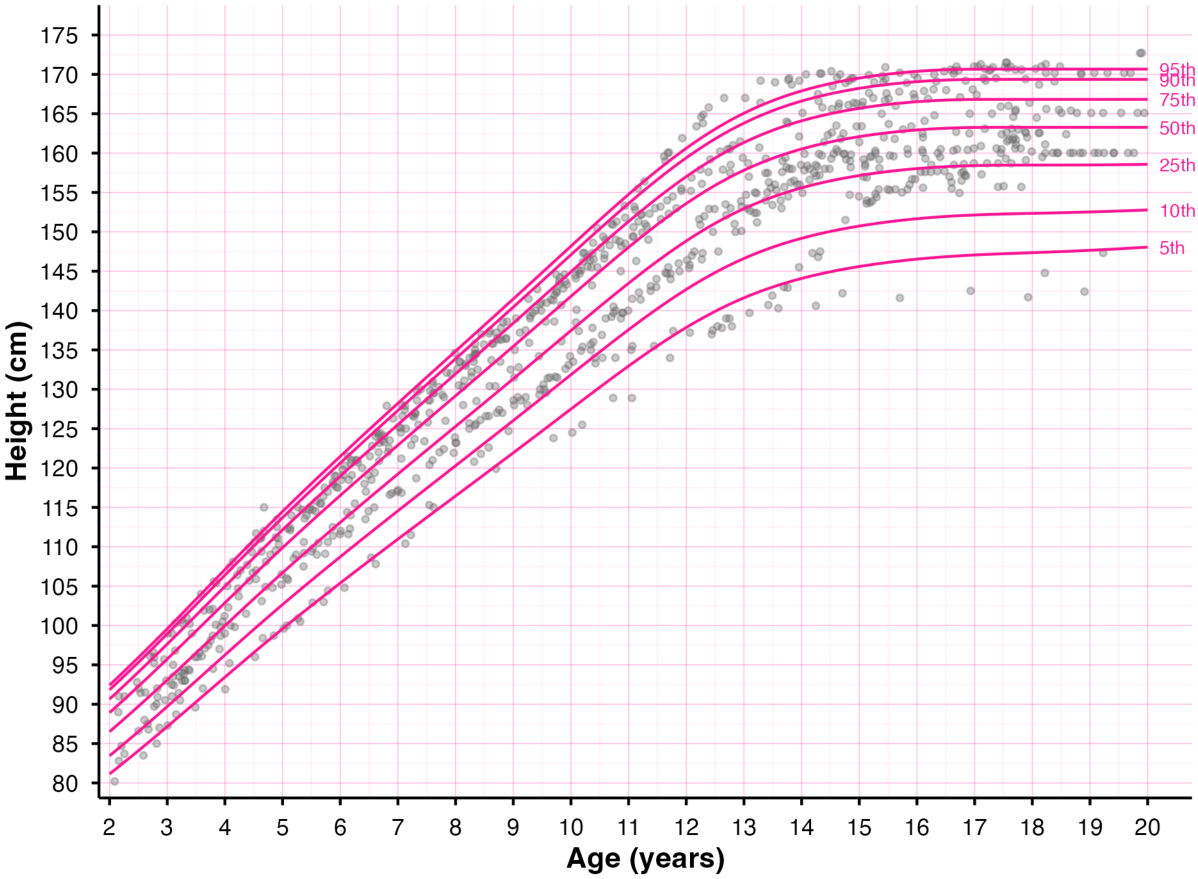

age_bin	N Observations
2-4	79
4-6	91
6-8	99
8-10	112
10-12	111
12-14	105
14-16	120
16-18	110
18-20	50

Supplementary Figure 4. Condition: Cystic Fibrosis, Sex: Female

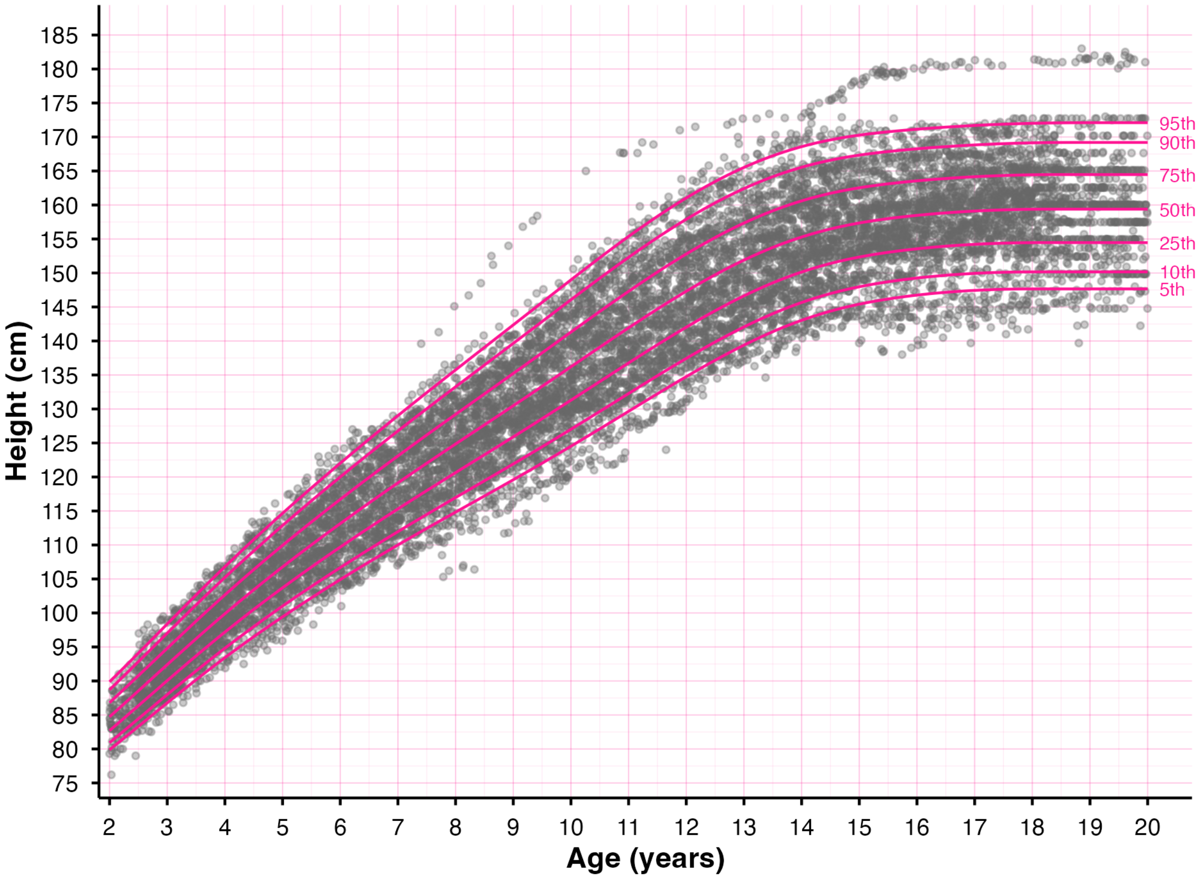

age_bin	N Observations
2-4	1,000
4-6	1,141
6-8	1,215
8-10	1,364
10-12	1,349
12-14	1,374
14-16	1,403
16-18	1,337
18-20	821

Supplementary Figure 5. Condition: Cystic Fibrosis, Sex: Male, Genotype: M/M

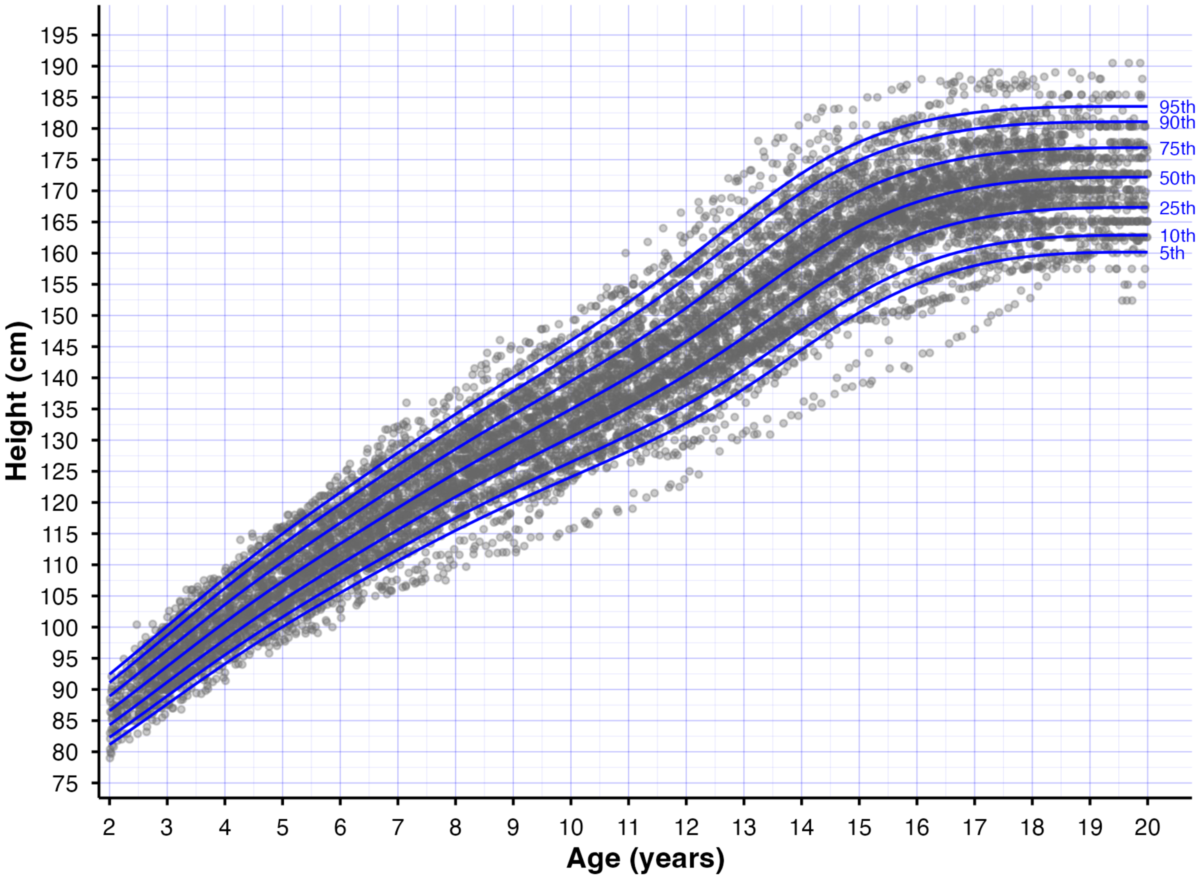

age_bin	N Observations
2-4	918
4-6	1,157
6-8	1,153
8-10	1,142
10-12	1,179
12-14	1,185
14-16	1,151
16-18	1,126
18-20	803

Supplementary Figure 6. Condition: Cystic Fibrosis, Sex: Male, Genotype: M/R

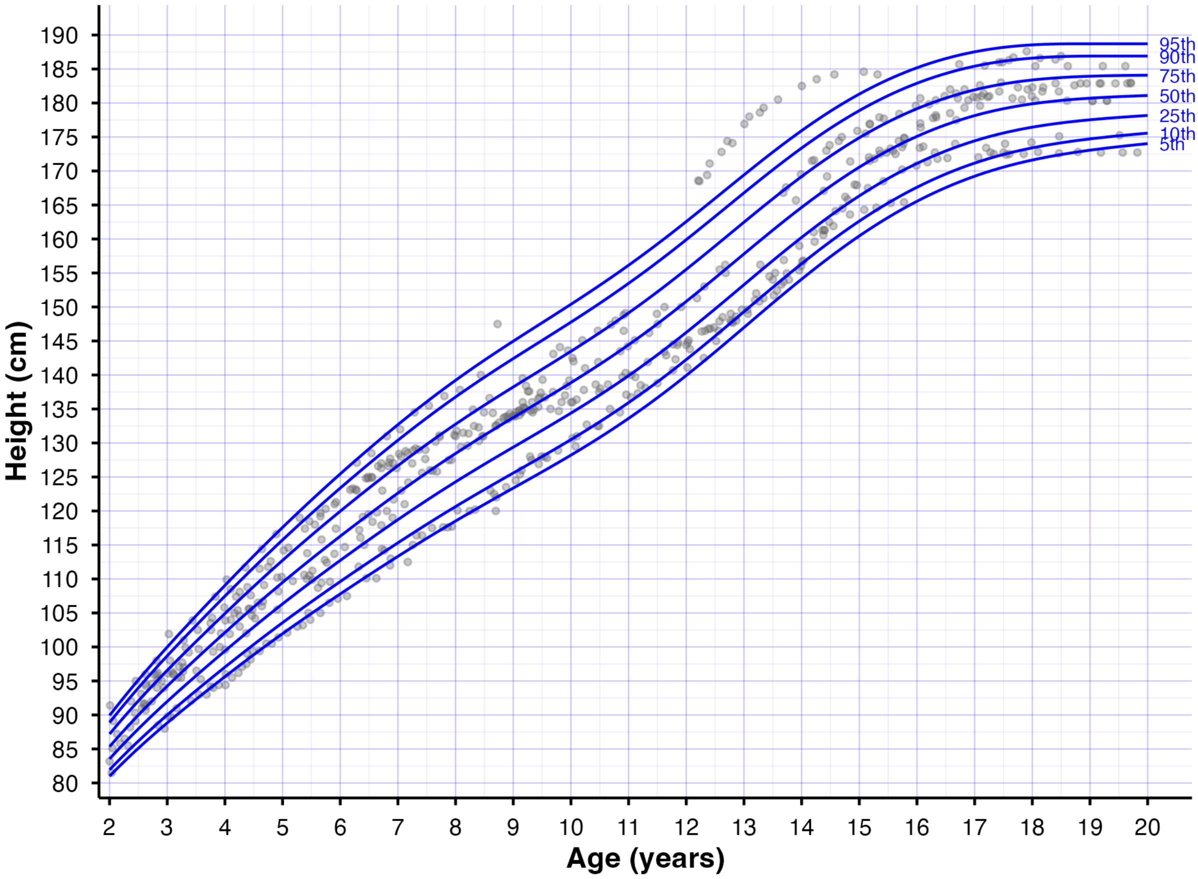

age_bin	N Observations
2-4	72
4-6	81
6-8	76
8-10	81
10-12	53
12-14	63
14-16	61
16-18	56
18-20	35

Supplementary Figure 7. Condition: Cystic Fibrosis, Sex: Male

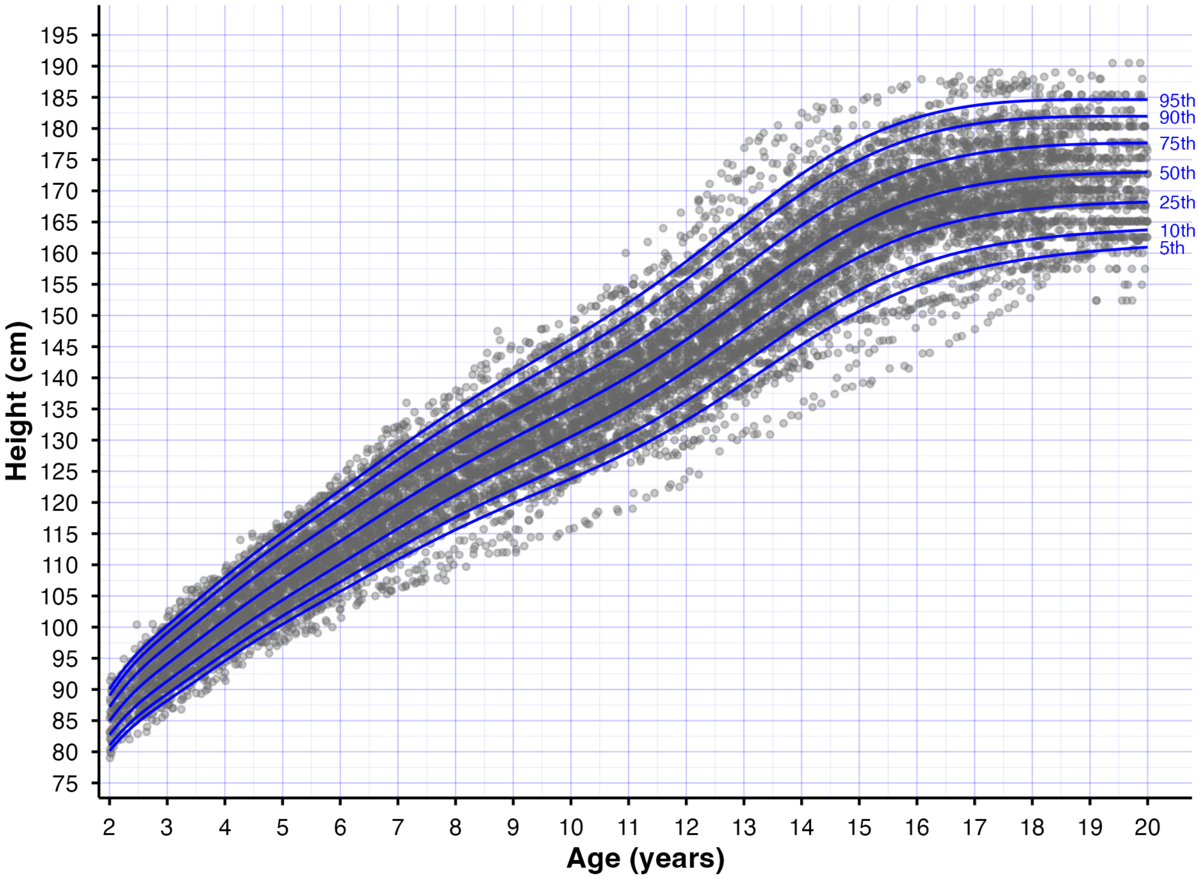

age_bin	N Observations
2-4	1,065
4-6	1,328
6-8	1,282
8-10	1,263
10-12	1,341
12-14	1,332
14-16	1,267
16-18	1,221
18-20	865

Supplementary Figure 8. Condition: DiGeorge Syndrome, Sex: Female

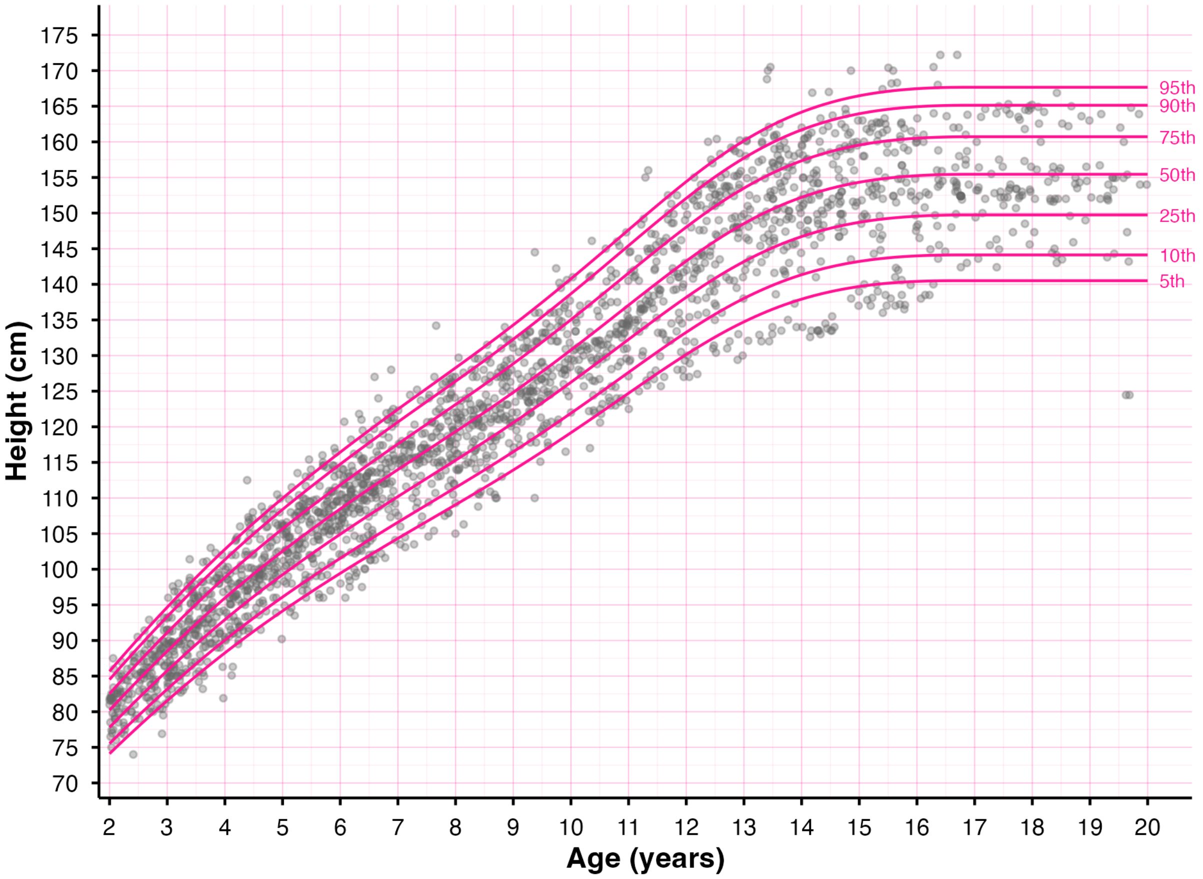

age_bin	N Observations
2-4	345
4-6	342
6-8	329
8-10	284
10-12	225
12-14	237
14-16	212
16-18	114
18-20	65

Supplementary Figure 9. Condition: Digeorge Syndrome, Sex: Male

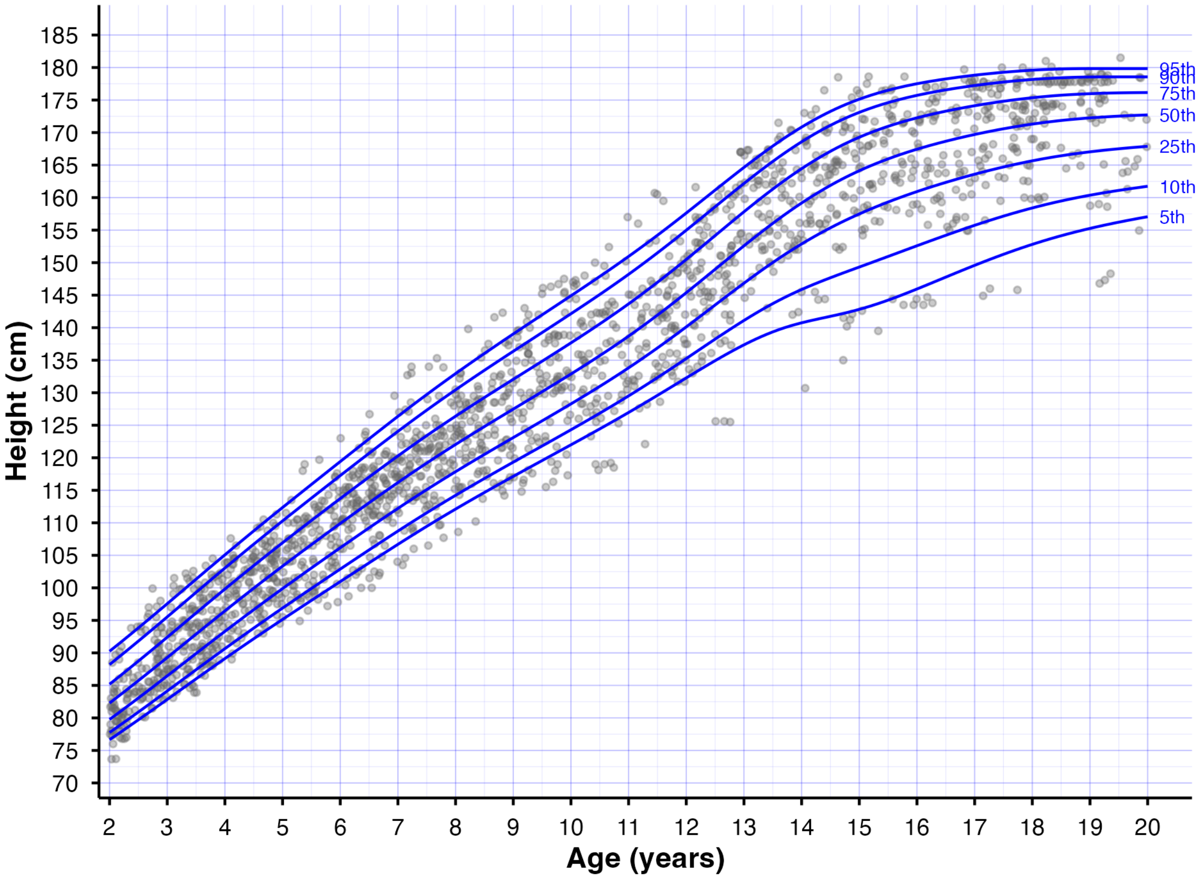

age_bin	N Observations
2-4	282
4-6	258
6-8	270
8-10	218
10-12	171
12-14	191
14-16	160
16-18	155
18-20	103

Supplementary Figure 10. Condition: Down Syndrome, Sex: Female

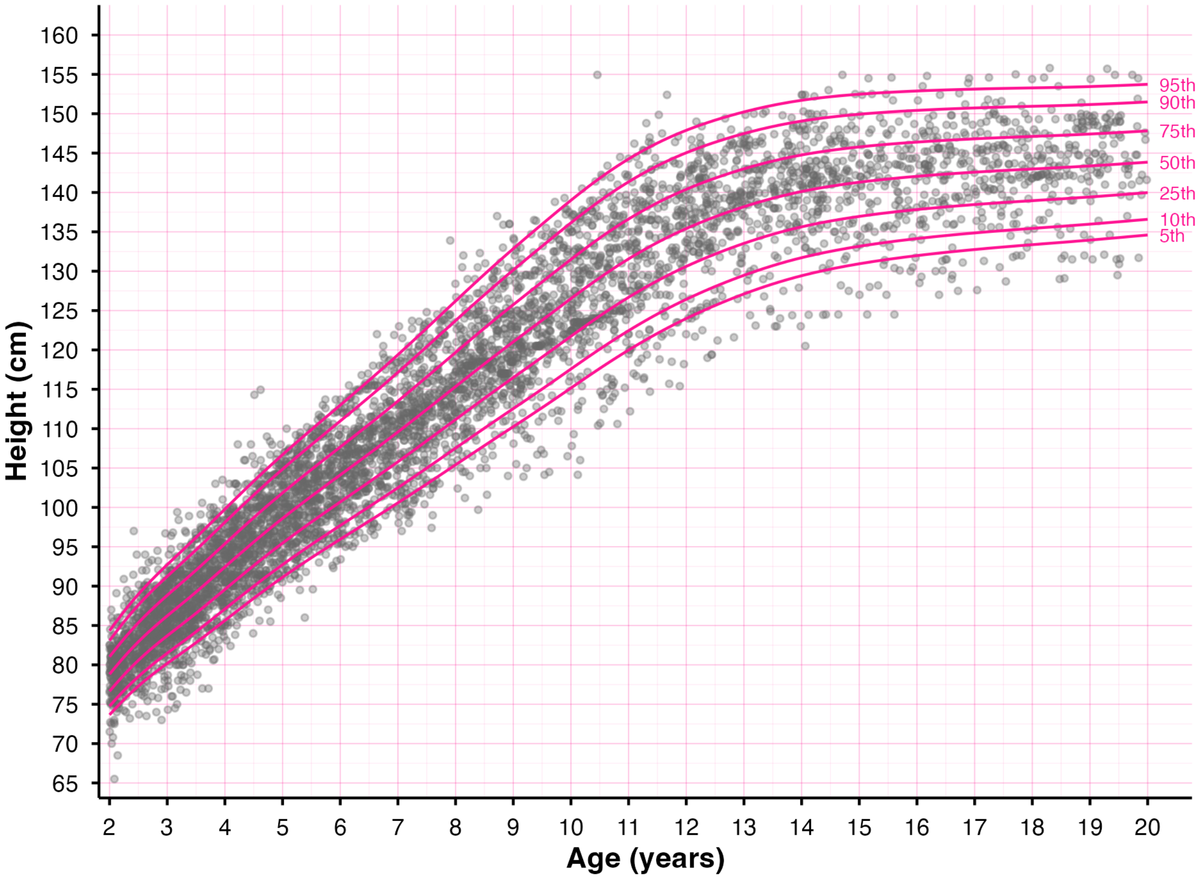

age_bin	N Observations
2-4	1,417
4-6	1,110
6-8	809
8-10	691
10-12	534
12-14	381
14-16	285
16-18	242
18-20	181

Supplementary Figure 11. Condition: Down Syndrome, Sex: Male

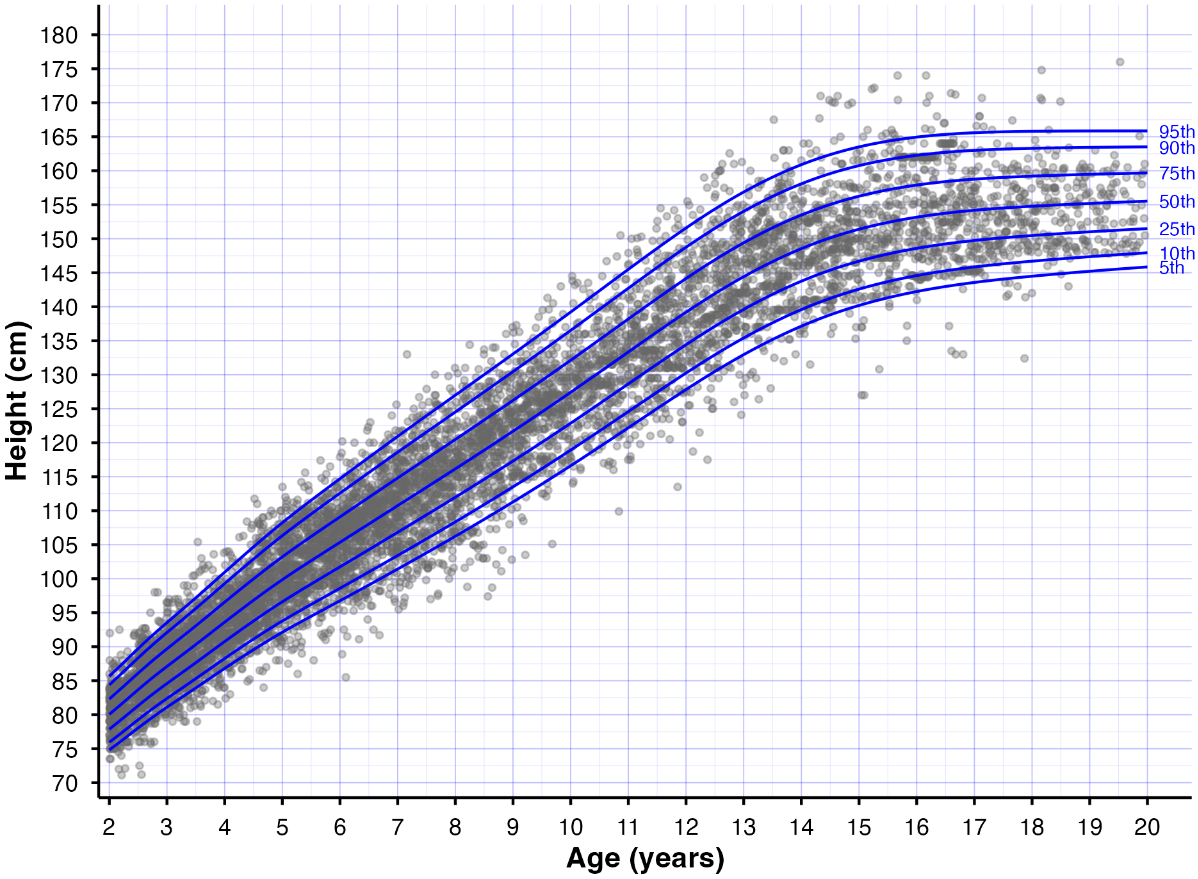

age_bin	N Observations
2-4	1,735
4-6	1,500
6-8	1,254
8-10	900
10-12	744
12-14	729
14-16	553
16-18	445
18-20	213

Supplementary Figure 12. Condition: Duchenne Muscular Dystrophy, Sex: Male

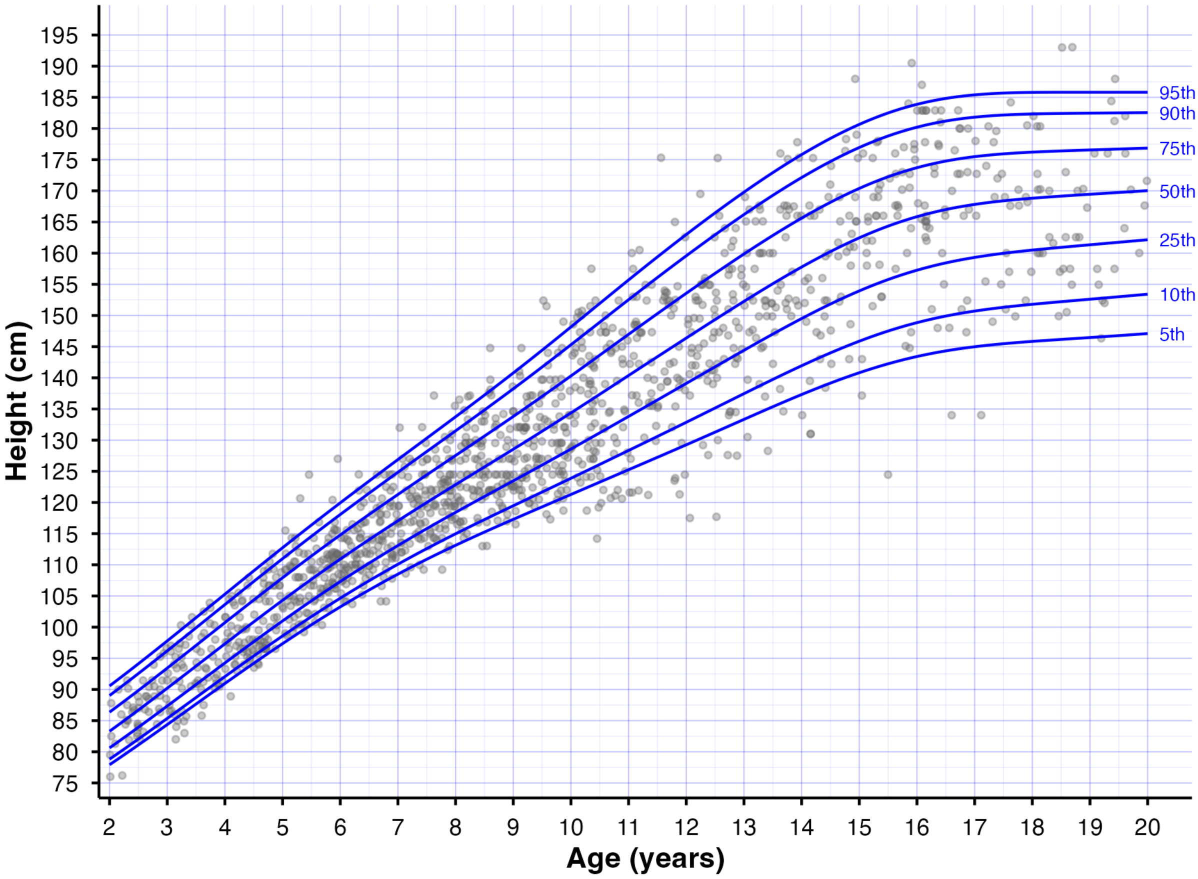

age_bin	N Observations
2-4	112
4-6	204
6-8	230
8-10	233
10-12	173
12-14	143
14-16	98
16-18	75
18-20	45

Supplementary Figure 13. Condition: Fragile X Syndrome, Sex: Male

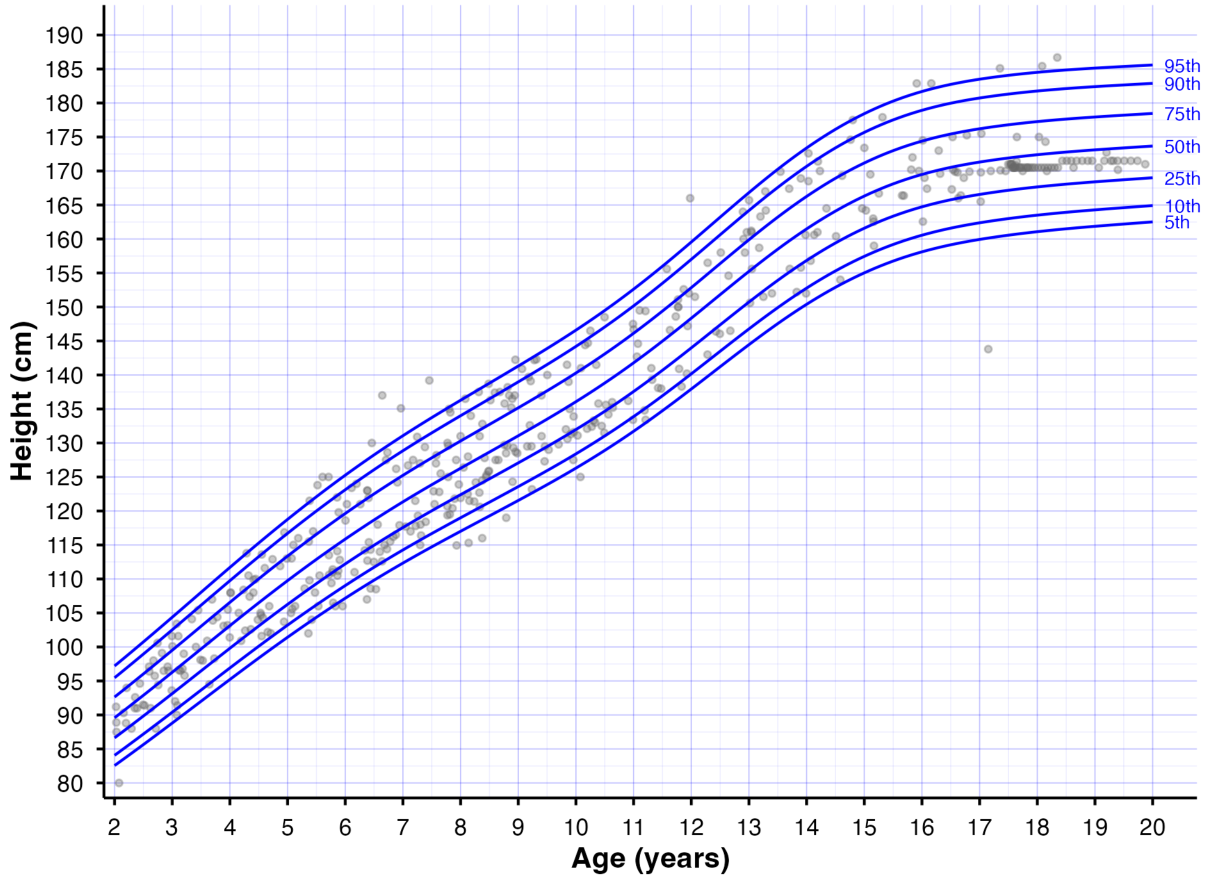

age_bin	N Observations
2-4	54
4-6	61
6-8	67
8-10	69
10-12	46
12-14	31
14-16	28
16-18	51
18-20	31

Supplementary Figure 14. Condition: Hemophilia A, Sex: Male

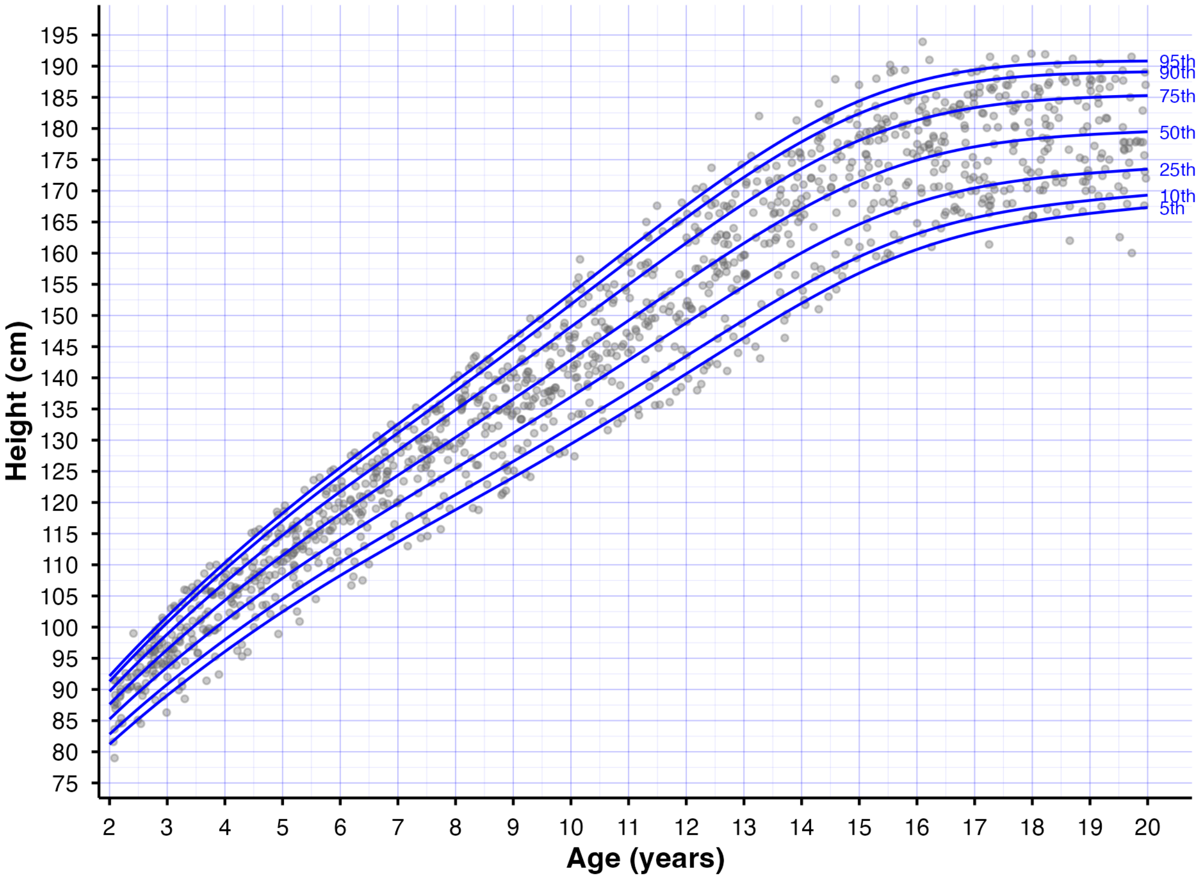

age_bin	N Observations
2-4	153
4-6	145
6-8	139
8-10	152
10-12	131
12-14	142
14-16	119
16-18	139
18-20	110

Supplementary Figure 15. Condition: Hemophilia B, Sex: Male

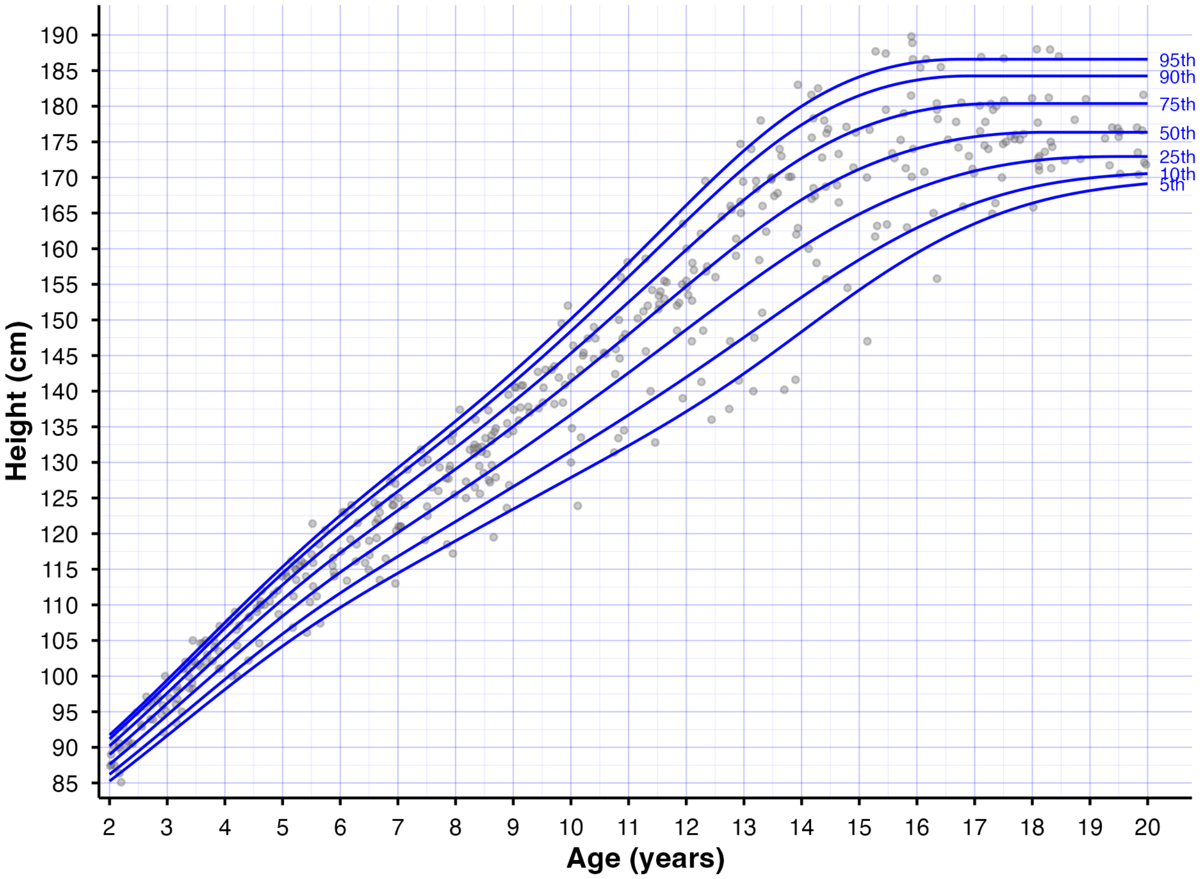

age_bin	N Observations
2-4	62
4-6	49
6-8	53
8-10	55
10-12	47
12-14	52
14-16	43
16-18	39
18-20	32

Supplementary Figure 16. Condition: Klinefelter, Sex: Male

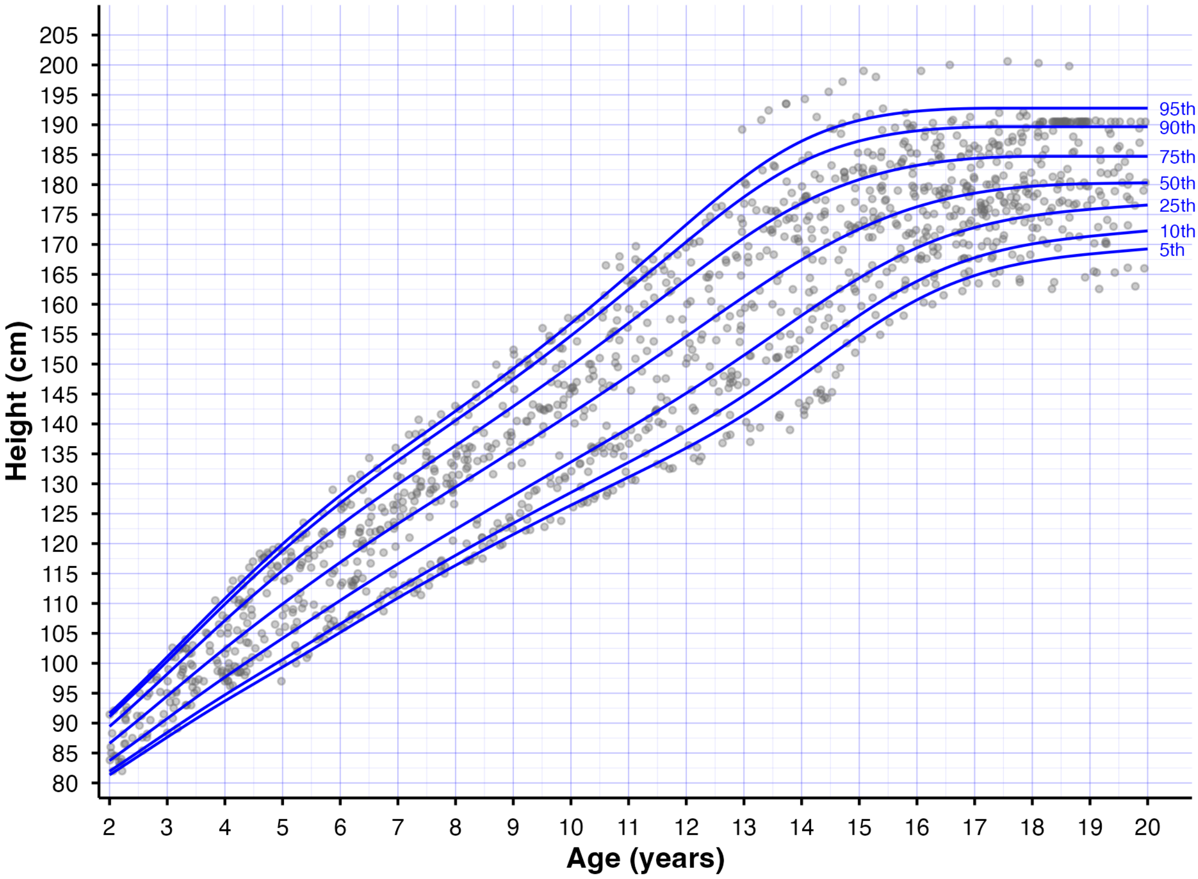

age_bin	N Observations
2-4	97
4-6	137
6-8	145
8-10	125
10-12	136
12-14	137
14-16	180
16-18	188
18-20	141

Supplementary Figure 17. Condition: Marfan Syndrome, Sex: Female

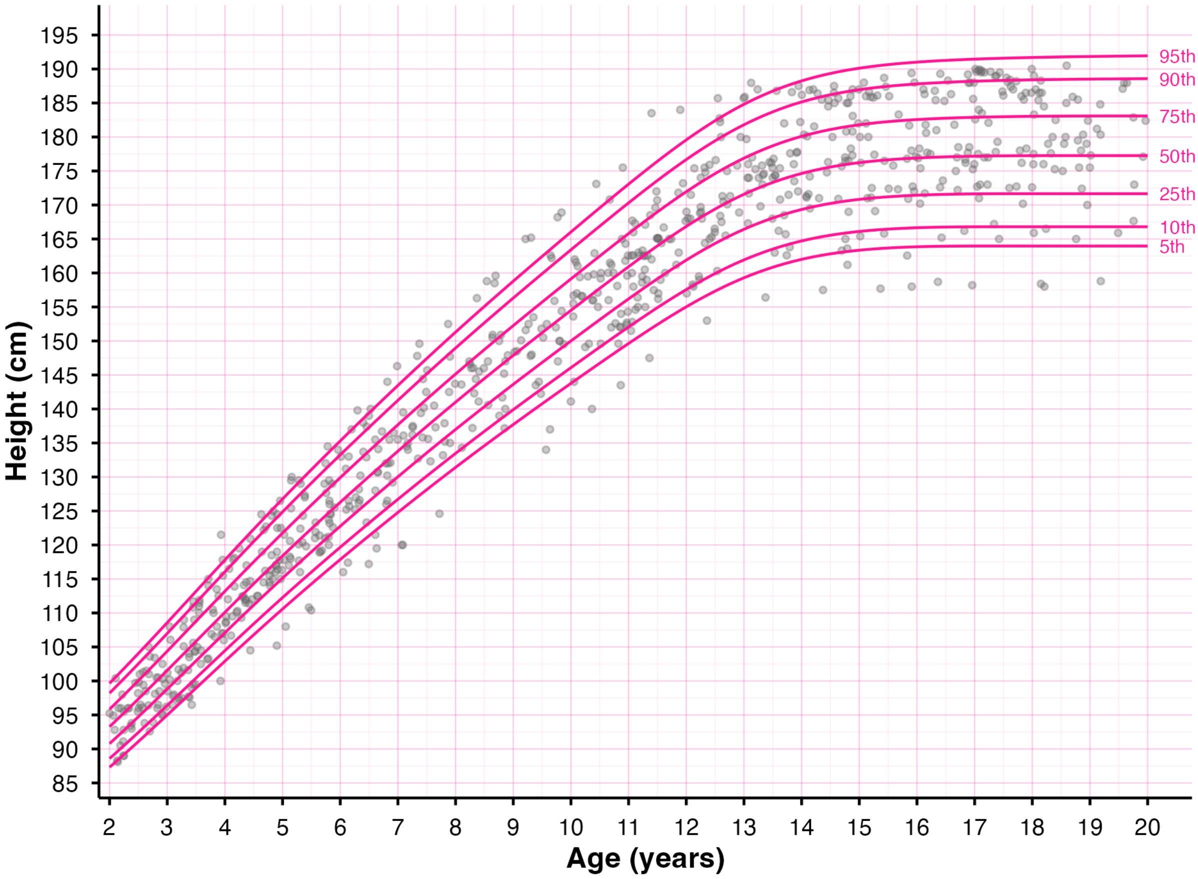

age_bin	N Observations
2-4	110
4-6	104
6-8	76
8-10	59
10-12	98
12-14	88
14-16	82
16-18	86
18-20	51

Supplementary Figure 18. Condition: Marfan Syndrome, Sex: Male

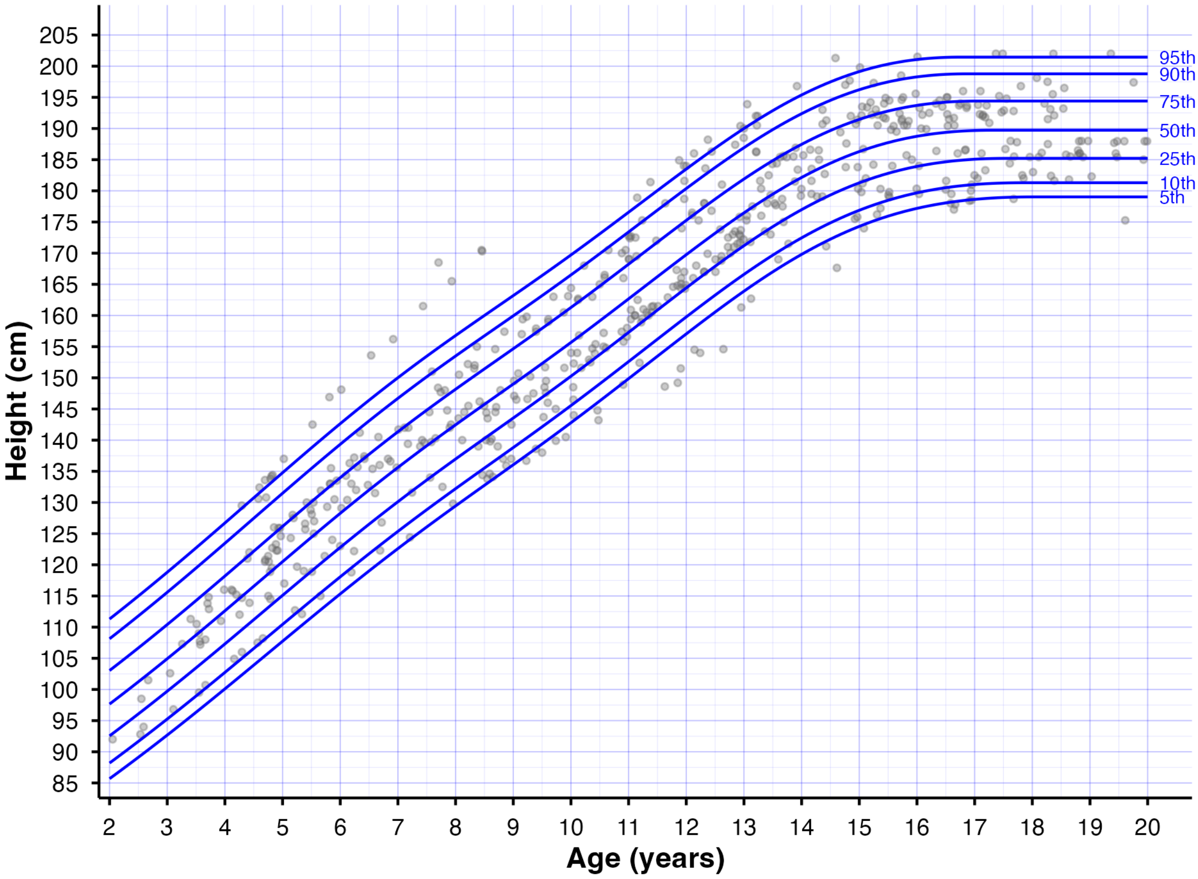

age_bin	N Observations
2-4	21
4-6	67
6-8	51
8-10	63
10-12	81
12-14	80
14-16	72
16-18	66
18-20	39

Supplementary Figure 19. Condition: Myotonic Dystrophy 1, Sex: Female

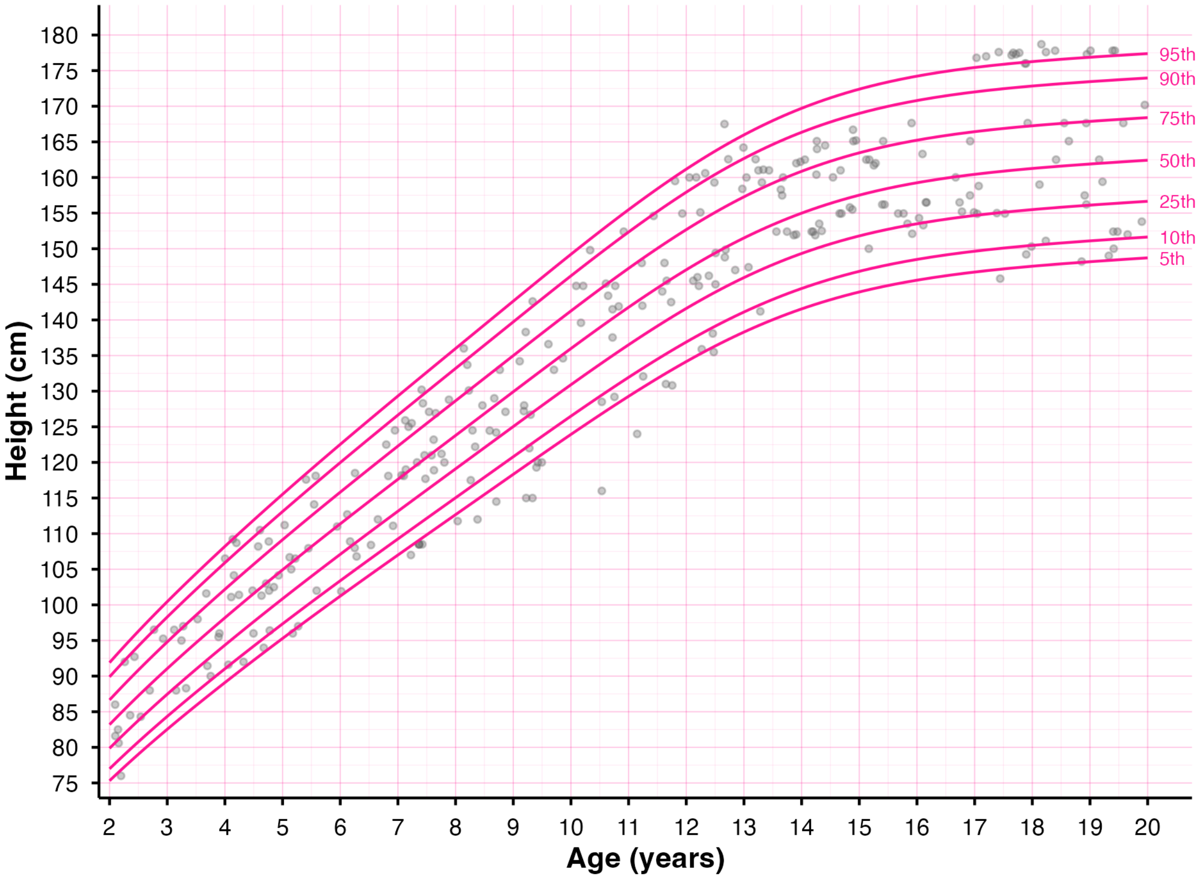

age_bin	N Observations
2-4	23
4-6	32
6-8	36
8-10	30
10-12	27
12-14	38
14-16	32
16-18	28
18-20	26

Supplementary Figure 20. Condition: Neurofibromatosis Type 1, Sex: Female

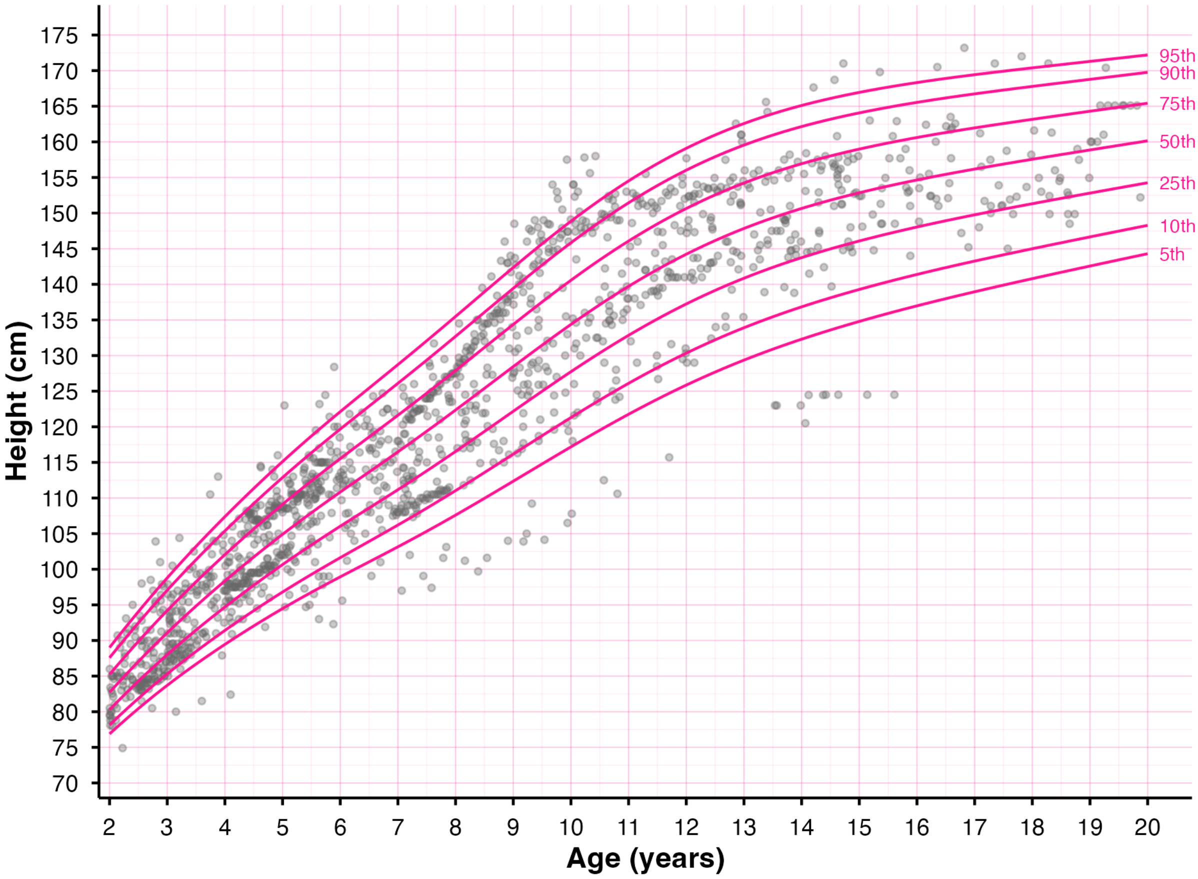

age_bin	N Observations
2-4	257
4-6	307
6-8	201
8-10	199
10-12	144
12-14	115
14-16	79
16-18	41
18-20	31

Supplementary Figure 21. Condition: Neurofibromatosis Type 1, Sex: Male

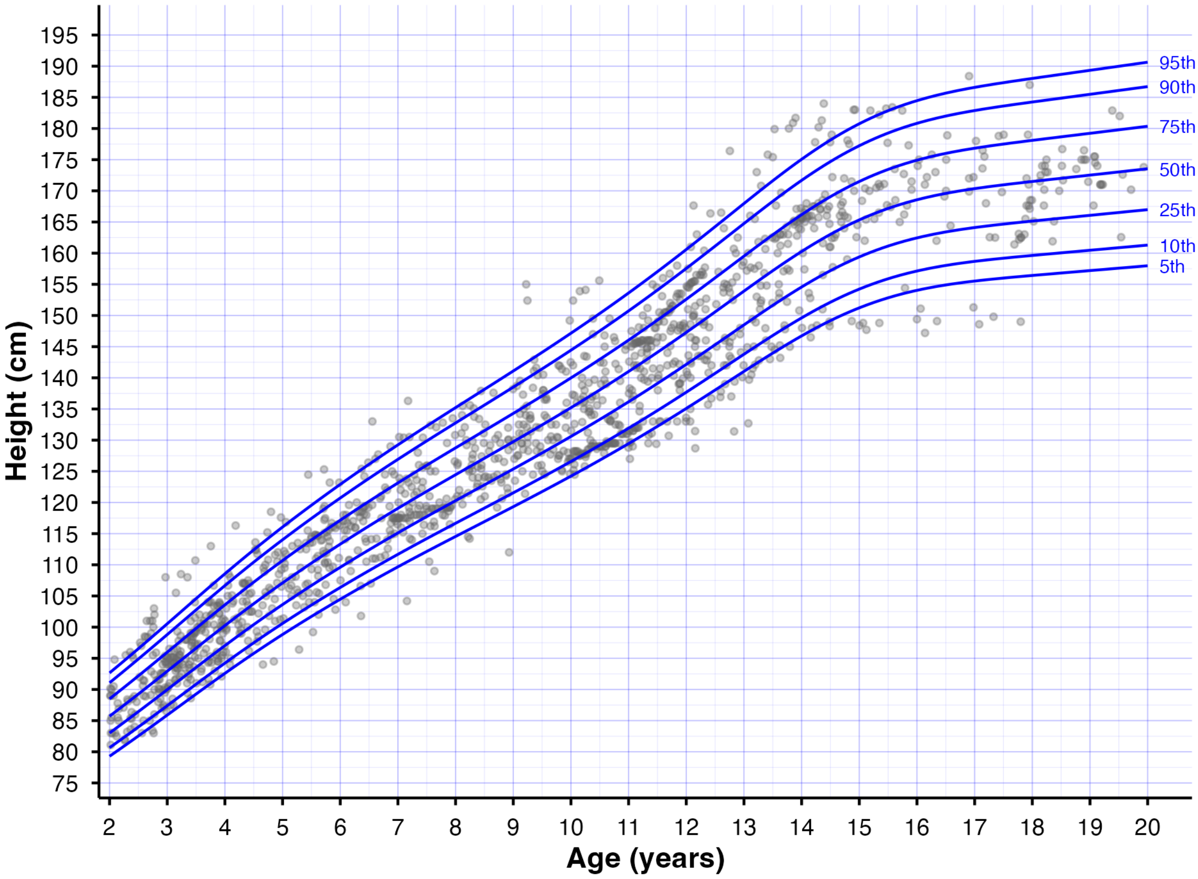

age_bin	N Observations
2-4	238
4-6	158
6-8	161
8-10	158
10-12	245
12-14	177
14-16	103
16-18	50
18-20	37

Supplementary Figure 22. Condition: Prader Willi Syndrome, Sex: Male

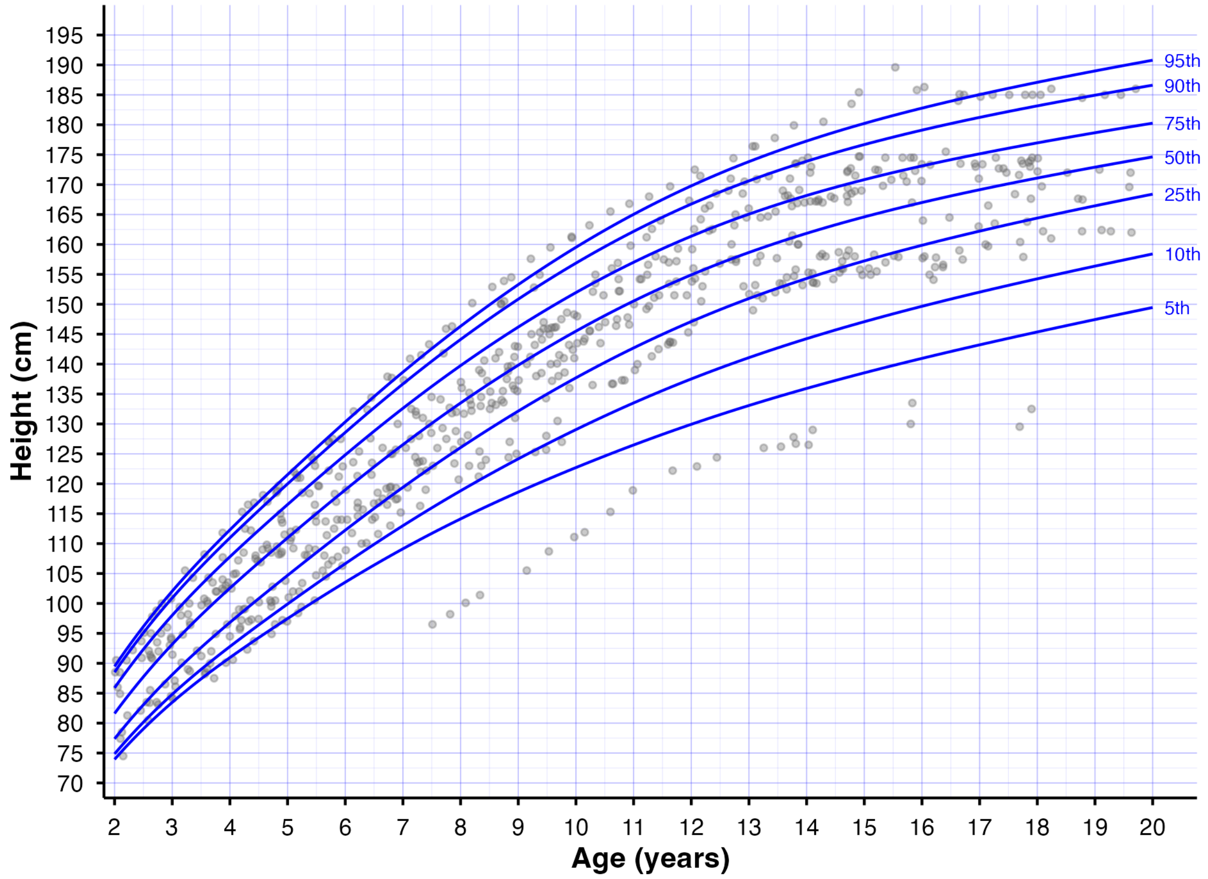

age_bin	N Observations
2-4	82
4-6	109
6-8	83
8-10	97
10-12	68
12-14	79
14-16	71
16-18	51
18-20	21

Supplementary Figure 23. Condition: Turner Syndrome, Sex: Female

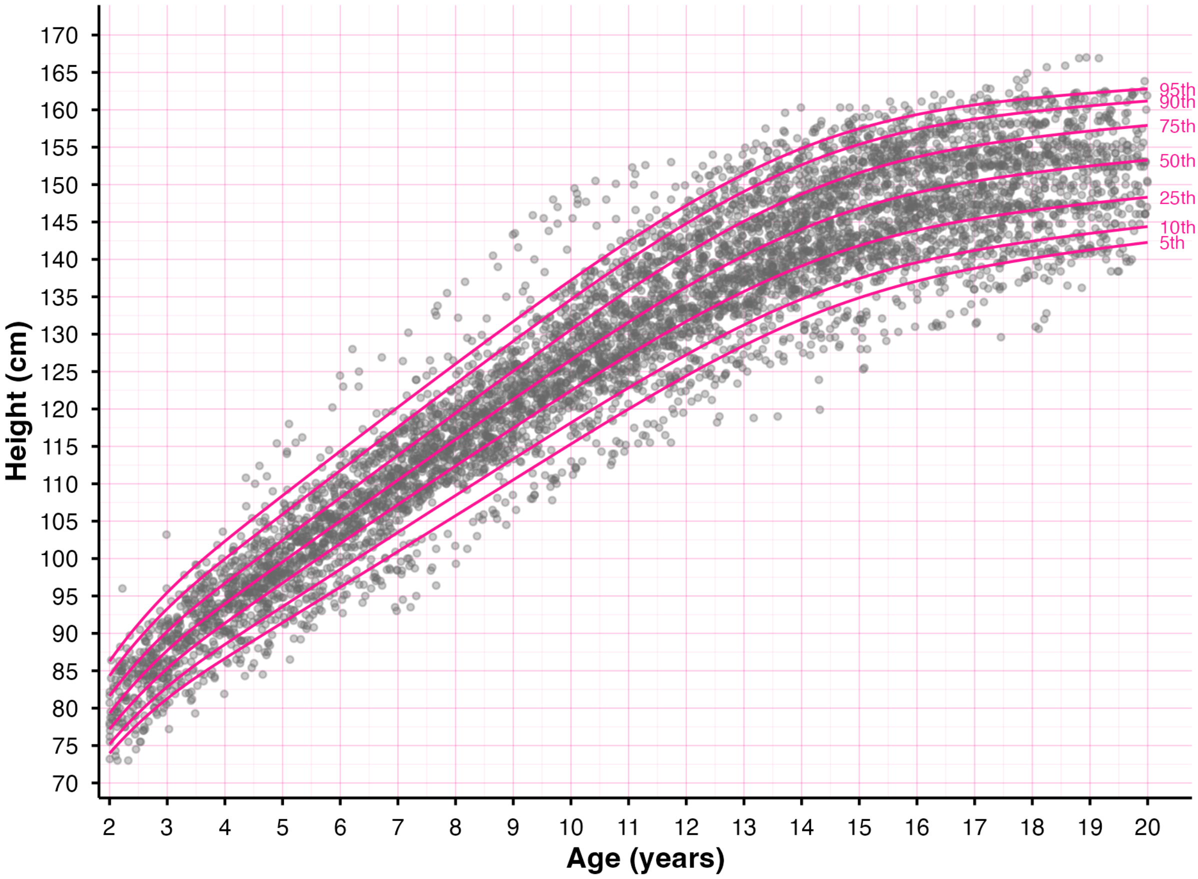

age_bin	N Observations
2-4	461
4-6	657
6-8	694
8-10	734
10-12	810
12-14	822
14-16	834
16-18	692
18-20	450

Supplementary Figure 24. Condition: Unaffected, Sex: Female

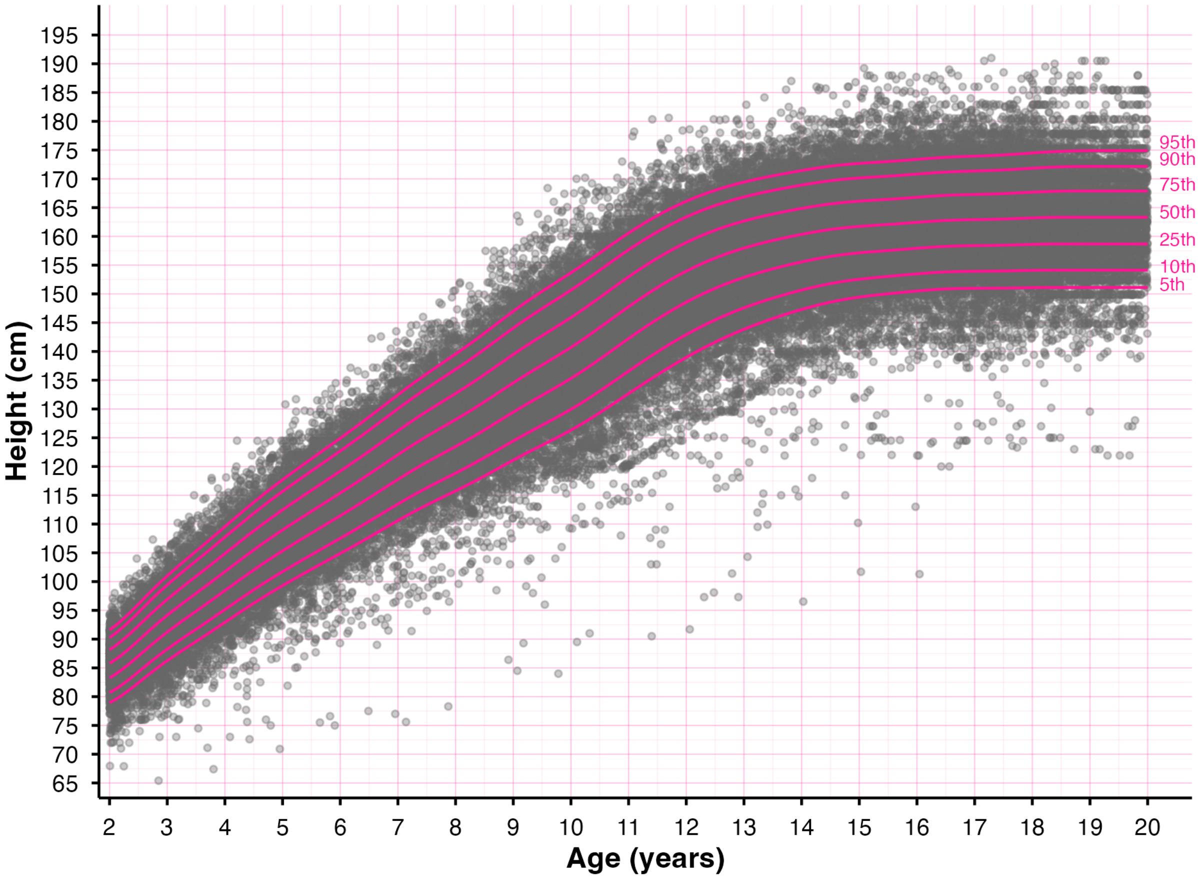

age_bin	N Observations
2-4	9,902
4-6	9,273
6-8	9,101
8-10	9,569
10-12	10,212
12-14	11,550
14-16	13,252
16-18	13,766
18-20	7,675

Supplementary Figure 25. Condition: Unaffected, Sex: Male

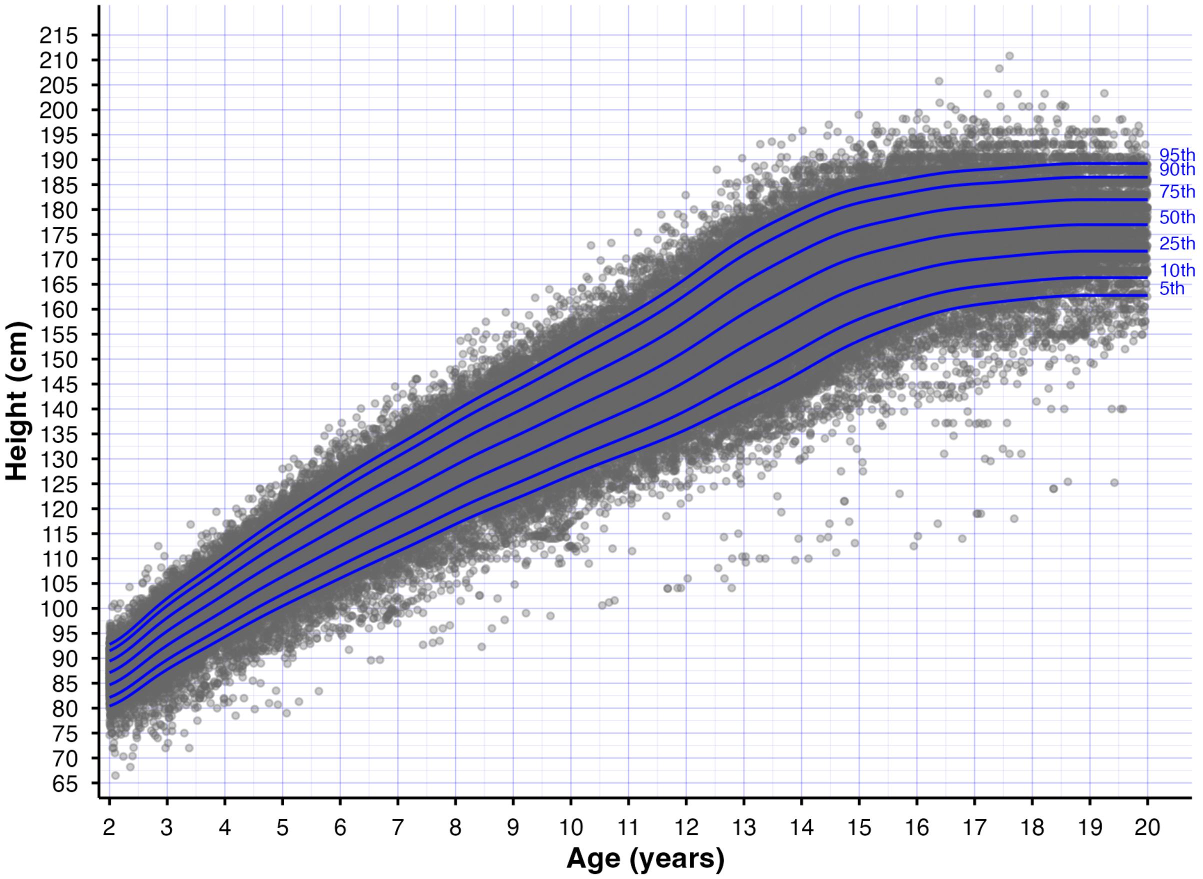

age_bin	N Observations
2-4	11,360
4-6	11,433
6-8	10,909
8-10	10,455
10-12	10,678
12-14	11,425
14-16	11,422
16-18	10,128
18-20	5,190

Supplementary Figure 26. Condition: Williams Beuren Syndrome, Sex: Male

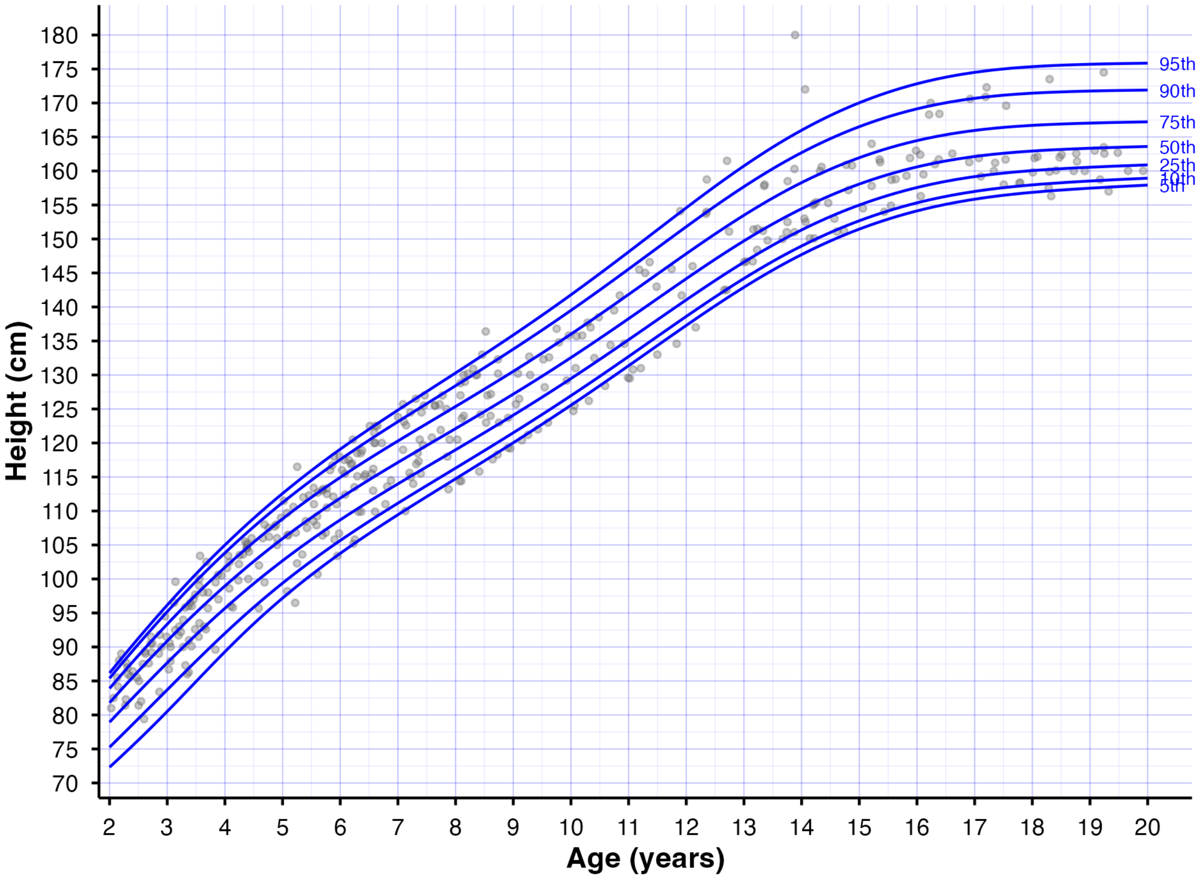

age_bin	N Observations
2-4	72
4-6	68
6-8	63
8-10	45
10-12	28
12-14	26
14-16	29
16-18	22
18-20	23

Supplementary Table 1. Growth-related Human Phenotype Ontology (HPO) terms in Online Mendelian Inheritance in Man (OMIM) and Orphanet.

HPO_ID	term_id	name	Include in study	Size	Intensity	Timing
HP:0000839	839	Pituitary dwarfism	0	Shorter	Unknown	Unknown
HP:0003498	3498	Disproportionate short stature	0	Shorter	Unknown	Unknown
HP:0003502	3502	Mild short stature	0	Shorter	Unknown	Unknown
HP:0003508	3508	Proportionate short stature	0	Shorter	Unknown	Unknown
HP:0003510	3510	Severe short stature	0	Shorter	Unknown	Unknown
HP:0003521	3521	Disproportionate short-trunk short stature	0	Shorter	Unknown	Unknown
HP:0004322	4322	Short stature	1	Shorter	Unknown	Unknown
HP:0005026	5026	Mesomelic/rhizomelic limb shortening	0	Shorter	Unknown	Unknown
HP:0005069	5069	Rhizo-meso-acromelic limb shortening	0	Shorter	Unknown	Unknown
HP:0008845	8845	Mesomelic short stature	0	Shorter	Unknown	Unknown
HP:0008848	8848	Moderately short stature	0	Shorter	Unknown	Unknown
HP:0008857	8857	Neonatal short-trunk short stature	0	Shorter	Unknown	Unknown
HP:0008873	8873	Disproportionate short-limb short stature	0	Shorter	Unknown	Unknown
HP:0008890	8890	Severe short-limb dwarfism	0	Shorter	Unknown	Unknown
HP:0008905	8905	Rhizomelia	0	Shorter	Unknown	Unknown
HP:0008909	8909	Lethal short-limbed short stature	0	Shorter	Unknown	Unknown
HP:0008921	8921	Neonatal short-limb short stature	0	Shorter	Unknown	Unknown
HP:0008922	8922	Childhood-onset short-trunk short stature	0	Shorter	Unknown	Unknown
HP:0008929	8929	Asymmetric short stature	0	Shorter	Unknown	Unknown
HP:0011404	11404	Lethal short-trunk short stature	0	Shorter	Unknown	Unknown
HP:0011405	11405	Childhood onset short-limb short stature	0	Shorter	Unknown	Unknown
HP:0011406	11406	Infancy onset short-trunk short stature	0	Shorter	Unknown	Unknown
HP:0012106	12106	Rhizomelic leg shortening	0	Shorter	Unknown	Unknown
HP:0003517	3517	Birth length greater than 97th percentile	0	Shorter	Unknown	Unknown
HP:0003561	3561	Birth length less than 3rd percentile	0	Shorter	Unknown	Unknown

HP:0000098	98	Tall stature	1	Taller	Unknown	Unknown
HP:0001519	1519	Disproportionate tall stature	1	Taller	Unknown	Unknown
HP:0001533	1533	Slender build	0	Taller	Unknown	Unknown
HP:0001548	1548	Overgrowth	1	Taller	Unknown	Unknown
HP:0003782	3782	Eunuchoid habitus	0	Taller	Unknown	Unknown
HP:0011407	11407	Proportionate tall stature	0	Taller	Unknown	Unknown
HP:0000002	2	Abnormality of body height	0	Both	Unknown	Unknown
HP:0001510	1510	Growth delay	1	Unknown	Lower	Unknown
HP:0001530	1530	Mild postnatal growth retardation	0	Unknown	Lower	Unknown
HP:0008850	8850	Severe postnatal growth retardation	0	Unknown	Lower	Unknown
HP:0008855	8855	Moderate postnatal growth retardation	0	Unknown	Lower	Unknown
HP:0008897	8897	Postnatal growth retardation	1	Unknown	Lower	Unknown
HP:0012569	12569	Delayed menarche	0	Unknown	Unknown	Late (female only)
HP:0000823	823	Delayed puberty	1	Unknown	Unknown	Late
HP:0031087	31087	Absent pubertal growth spurt	0	Unknown	Unknown	Late
HP:0025453	25453	Delayed adrenarche	0	Unknown	Unknown	Late
HP:0008185	8185	Precocious puberty in males	0	Unknown	Unknown	Early (male only)
HP:0010465	10465	Precocious puberty in females	0	Unknown	Unknown	Early (female only)
HP:0000826	826	Precocious puberty	1	Unknown	Unknown	Early
HP:0008204	8204	Precocious puberty with Sertoli cell tumor	0	Unknown	Unknown	Early
HP:0008236	8236	Isosexual precocious puberty	0	Unknown	Unknown	Early
HP:0012412	12412	Premature adrenarche	1	Unknown	Unknown	Early
HP:0100000	100000	Early onset of sexual maturation	1	Unknown	Unknown	Early
HP:0004991	4991	Rhizomelic arm shortening	0	-	-	-
HP:0001507	1507	Growth abnormality	0	-	-	-
HP:0001508	1508	Failure to thrive	0	-	-	-
HP:0001525	1525	Severe failure to thrive	0	-	-	-
HP:0001531	1531	Failure to thrive in infancy	0	-	-	-
HP:0008866	8866	Failure to thrive secondary to recurrent infections	0	-	-	-
HP:0045081	45081	Abnormality of body mass index	0	-	-	-
HP:0001511	1511	Intrauterine growth retardation	0	-	-	-

HP:0001513	1513	Obesity	0	-	-	-
HP:0001518	1518	Small for gestational age	0	-	-	-
HP:0001520	1520	Large for gestational age	0	-	-	-
HP:0001528	1528	Hemihypertrophy	0	-	-	-
HP:0001824	1824	Weight loss	0	-	-	-
HP:0001956	1956	Truncal obesity	0	-	-	-
HP:0003363	3363	Abdominal situs inversus	0	-	-	-
HP:0004323	4323	Abnormality of body weight	0	-	-	-
HP:0004324	4324	Increased body weight	0	-	-	-
HP:0004325	4325	Decreased body weight	0	-	-	-
HP:0004326	4326	Cachexia	0	-	-	-
HP:0008846	8846	Severe intrauterine growth retardation	0	-	-	-
HP:0008883	8883	Mild intrauterine growth retardation	0	-	-	-
HP:0008915	8915	Childhood-onset truncal obesity	0	-	-	-
HP:0011408	11408	Moderate intrauterine growth retardation	0	-	-	-
HP:0011536	11536	Right atrial isomerism	0	-	-	-
HP:0011537	11537	Left atrial isomerism	0	-	-	-
HP:0011620	11620	Abnormality of abdominal situs	0	-	-	-
HP:0012743	12743	Abdominal obesity	0	-	-	-
HP:0012772	12772	Abnormal upper to lower segment ratio	0	-	-	-
HP:0012773	12773	Reduced upper to lower segment ratio	0	-	-	-
HP:0012774	12774	Increased upper to lower segment ratio	0	-	-	-
HP:0025499	25499	Class I obesity	0	-	-	-
HP:0025500	25500	Class II obesity	0	-	-	-
HP:0025501	25501	Class III obesity	0	-	-	-
HP:0025502	25502	Overweight	0	-	-	-
HP:0025515	25515	Delayed thelarche	0	-	-	-
HP:0025521	25521	Increased body fat percentage	0	-	-	-
HP:0030853	30853	Heterotaxy	0	-	-	-
HP:0031418	31418	Increased body mass index	0	-	-	-
HP:0031564	31564	Bronchial isomerism	0	-	-	-
HP:0031565	31565	Abdominal situs ambiguus	0	-	-	-
HP:0031818	31818	Abnormal waist to hip ratio	0	-	-	-

HP:0031819	31819	Increased waist to hip ratio	0	-	-	-
HP:0031820	31820	Decreased waist to hip ratio	0	-	-	-
HP:0031853	31853	Isomerism	0	-	-	-
HP:0031854	31854	Left Isomerism	0	-	-	-
HP:0031855	31855	Right isomerism	0	-	-	-
HP:0033170	33170	Abnormal skinfold thickness measurement	0	-	-	-
HP:0033171	33171	Abnormal triceps skinfold thickness	0	-	-	-
HP:0033172	33172	Increased triceps skinfold thickness	0	-	-	-
HP:0033794	33794	Acral overgrowth	0	-	-	-
HP:0033795	33795	Growth without growth hormone	0	-	-	-
HP:0041079	41079	Decreased body fat percentage	0	-	-	-
HP:0045082	45082	Decreased body mass index	0	-	-	-
HP:0100553	100553	Hemihypertrophy of lower limb	0	-	-	-
HP:0100554	100554	Hemihypertrophy of upper limb	0	-	-	-
HP:0100555	100555	Asymmetric growth	0	-	-	-
HP:0100556	100556	Hemiatrophy	0	-	-	-
HP:0100557	100557	Hemiatrophy of lower limb	0	-	-	-
HP:0100558	100558	Hemiatrophy of upper limb	0	-	-	-
HP:0100559	100559	Lower limb asymmetry	0	-	-	-
HP:0100560	100560	Upper limb asymmetry	0	-	-	-
HP:0200053	200053	Hemihypotrophy of lower limb	0	-	-	-

Supplementary Table 2. Height-related Human Phenotype Ontology (HPO) terms in Online Mendelian Inheritance in Man (OMIM) and Orphanet by disease.

					Height-related terms from knowledgebases (OMIM and Orphanet)						Height attributes		
**OMIM ID**	**OrphaCode**	**Disease name**	**OMIM ID**	**OrphaCode**	**# total growth terms**	**# in both**	**# OMIM growth terms**	**# Orphanet growth terms**	**HPO term ID(s)**	**HPO term name(s)**	**Size**	**Intensity**	**Timing**
154700	558	Marfan syndrome	154700	558	2	1	2	1	HP:0001519, HP:0000098	Disproportionate tall stature, Tall stature	Taller	Typical	Typical
162200	636	Neurofibromatosis, type 1	162200	636	5	1	2	4	HP:0004322, HP:0001548, HP:0000098, HP:0000823, HP:0000826,	Short stature, Overgrowth, Tall stature, Delayed puberty, Precocious puberty	Taller/Shorter	Typical	Later/Earlier
176270	739	Prader-Willi syndrome	176270	739	5	3	3	5	HP:0000823, HP:0000826, HP:0004322, HP:0001510, HP:0012412	Delayed puberty, Precocious puberty, Short stature, Growth delay, Premature adrenarche	Shorter	Lower	Later/Earlier
188400	567	DiGeorge syndrome	188400	567	1	1	1	1	HP:0004322	Short stature	Shorter	Typical	Typical
190685	870	Down syndrome	190685	870	2	1	1	2	HP:0004322, HP:0000823	Short stature, Delayed puberty	Shorter	Typical	Later
194050	904	Williams-Beuren syndrome	194050	904	3	1	2	2	HP:0004322, HP:0100000, HP:0000826,	Short stature, Early onset of sexual maturation, Precocious puberty	Shorter	Typical	Earlier

-	881	Turner syndrome	NA	881	4	-	-	4	HP:0000823, HP:0001510, HP:0004322, HP:0008897	Delayed puberty, Growth delay, Short stature, Postnatal growth retardation	Shorter	Lower	Later
219700	586	Cystic fibrosis	219700	586	0	0	0	0	-	-	Typical	Typical	Typical
310200	98896	Duchenne muscular dystrophy	310200	98896	0	0	0	0	-	-	Typical	Typical	Typical
306900	98879	Hemophilia B	306900	98879	0	0	0	0	-	-	Typical	Typical	Typical
306700	98878	Hemophilia A	306700	98878	0	0	0	0	-	-	Typical	Typical	Typical
118220	101081	Charcot-Marie-Tooth disease, type 1A	118220	101081	0	0	0	0	-	-	Typical	Typical	Typical
-	-	XXY syndrome (Kleinfelter)	NA	NA	-	-	-	-	-	-	-	-	-

Supplementary Table 3. STROBE Statement—checklist of items that should be included in reports of observational studies

	Item No.	Recommendation	Page No.	Relevant text from manuscript
**Title and abstract**	1	(*a*) Indicate the study's design with a commonly used term in the title or the abstract	2	See abstract
		(*b*) Provide in the abstract an informative and balanced summary of what was done and what was found	2	See abstract
**Introduction**				
Background/rationale	2	Explain the scientific background and rationale for the investigation being reported	3 – 4	The first and second paragraphs in the Introduction provide the scientific background and rationale.
Objectives	3	State specific objectives, including any prespecified hypotheses	4 – 5	The last paragraph in the Introduction provides the scientific objective of this study: to develop and apply an electronic health record (EHR)-based framework for modelling height in genetic conditions.
**Methods**				
Study design	4	Present key elements of study design early in the paper	11	The study design (single site, cohort study) is presented in the first paragraph of the Methods section (i.e., Step 1. Cohort Definition and Data Curation)
Setting	5	Describe the setting, locations, and relevant dates, including periods of recruitment, exposure, follow-up, and data collection	11 – 13	The setting, locations, and relevant dates, exposure, follow-up and data collection are presented in the first two subsections in the Methods section (i.e., Step 1. Cohort

				Definition and Data Curation, and Step 2. Data Cleaning and Preprocessing)
Participants	6	(*a*) *Cohort study*—Give the eligibility criteria, and the sources and methods of selection of participants. Describe methods of follow-up<br>*Case-control study*—Give the eligibility criteria, and the sources and methods of case ascertainment and control selection. Give the rationale for the choice of cases and controls<br>*Cross-sectional study*—Give the eligibility criteria, and the sources and methods of selection of participants	11 – 13	The eligibility criteria, sources and methods of selection of participants, and methods of follow-up are presented in the first two subsections in the Methods section (i.e., Step 1. Cohort Definition and Data Curation, and Step 2. Data Cleaning and Preprocessing)
		(*b*) *Cohort study*—For matched studies, give matching criteria and number of exposed and unexposed<br>*Case-control study*—For matched studies, give matching criteria and the number of controls per case	Not applicable. No matching was performed.	Not applicable. No matching was performed.
Variables	7	Clearly define all outcomes, exposures, predictors, potential confounders, and effect modifiers. Give diagnostic criteria, if applicable	12 – 13	The outcome (height) and exposures (age, sex) are defined in the second subsection in the Methods section (i.e., Step 2. Data Cleaning and Preprocessing)
Data sources/ measurement	8*	For each variable of interest, give sources of data and details of methods of assessment (measurement). Describe comparability of assessment methods if there is more than one group	11 – 13	The sources of data and details of measurement are provided in the first two subsections in the Methods section (i.e., Step 1. Cohort Definition and Data Curation, and Step 2. Data Cleaning and Preprocessing)
Bias	9	Describe any efforts to address potential sources of bias	12 – 13	To address potential measurement bias, we used a

				validated data cleaning algorithm and a model-based outlier detection approach. This information is provided in the second subsection in the Methods section (i.e., Step 2. Data Cleaning and Preprocessing)
Study size	10	Explain how the study size was arrived at	12 – 13	This information is provided in the second subsection in the Methods section (i.e., Step 2. Data Cleaning and Preprocessing)

Continued on next page

Quantitative variables	11	Explain how quantitative variables were handled in the analyses. If applicable, describe which groupings were chosen and why	13 – 16	The modelling approaches are described in Steps 3 – 6 in the Methods section.
Statistical methods	12	(*a*) Describe all statistical methods, including those used to control for confounding	13 – 16	All statistical methods are described in Steps 3 – 6 in the Methods section.
		(*b*) Describe any methods used to examine subgroups and interactions	13 – 16	Examinations of subgroups (e.g., unaffected vs. affected, male vs. female) are provided in Steps 3 – 6 in the Methods section.
		(*c*) Explain how missing data were addressed	12 – 13	No data imputation was performed. Analyses were based on available height measurements after data cleaning (Step 2).
		(*d*) *Cohort study*—If applicable, explain how loss to follow-up was addressed<br>*Case-control study*—If applicable, explain how matching of cases and controls was addressed<br>*Cross-sectional study*—If applicable, describe analytical methods taking account of sampling strategy	Not applicable. Participants were identified from electronic health records data with no active follow-up.	Not applicable. Participants were identified from electronic health records data with no active follow-up.
		(*e*) Describe any sensitivity analyses	13 – 15	Model selection and performance evaluation procedures are described in Steps 3 – 4.
**Results**				

Participants	13*	(a) Report numbers of individuals at each stage of study—eg numbers potentially eligible, examined for eligibility, confirmed eligible, included in the study, completing follow-up, and analysed	5	See Table 1
		(b) Give reasons for non-participation at each stage	Not applicable. Participants were identified from electronic health records data with no active recruitment.	Not applicable. Participants were identified from electronic health records data with no active recruitment.
		(c) Consider use of a flow diagram	5	See Table 1
Descriptive data	14*	(a) Give characteristics of study participants (eg demographic, clinical, social) and information on exposures and potential confounders	5	See Table 1
		(b) Indicate number of participants with missing data for each variable of interest	5	There was no missing data for the variables used in this study (e.g., age, sex). All analyses were based on available height measurements.
		(c) *Cohort study*—Summarise follow-up time (eg, average and total amount)	5	Follow-up time is provided in the first paragraph in the Results section.
Outcome data	15*	*Cohort study*—Report numbers of outcome events or summary measures over time	5	See table 1
		Case-control study—Report numbers in each exposure category, or summary measures of exposure	Not applicable. This is a longitudinal cohort study.	Not applicable. This is a longitudinal cohort study.

		Cross-sectional study—Report numbers of outcome events or summary measures	Not applicable. This is a longitudinal cohort study.	Not applicable. This is a longitudinal cohort study.
Main results	16	(*a*) Give unadjusted estimates and, if applicable, confounder-adjusted estimates and their precision (eg, 95% confidence interval). Make clear which confounders were adjusted for and why they were included	6 – 8	Unadjusted estimates and 95% confidence intervals are provided in the Results section.
		(*b*) Report category boundaries when continuous variables were categorized	Not applicable. Continuous variables were not categorized.	Not applicable. Continuous variables were not categorized.
		(*c*) If relevant, consider translating estimates of relative risk into absolute risk for a meaningful time period	Not applicable. No relative risk was reported.	Not applicable. No relative risk was reported.

Continued on next page

Other analyses	17	Report other analyses done—eg analyses of subgroups and interactions, and sensitivity analyses	7	A subgroup analysis on *CFTR* functional class was performed.
**Discussion**				
Key results	18	Summarise key results with reference to study objectives	8	Key study results with reference to study objectives were summarized in the first paragraph of the Discussion section.
Limitations	19	Discuss limitations of the study, taking into account sources of potential bias or imprecision. Discuss both direction and magnitude of any potential bias	10	Study limitations are described in the third paragraph in the Discussion section.
Interpretation	20	Give a cautious overall interpretation of results considering objectives, limitations, multiplicity of analyses, results from similar studies, and other relevant evidence	8 – 11	Interpretation of study results are provided in the first three paragraphs in the Discussion section.
Generalisability	21	Discuss the generalisability (external validity) of the study results	8 – 10	Generalizability of study results are provided in the first three paragraphs in the Discussion section.
**Other information**				
Funding	22	Give the source of funding and the role of the funders for the present study and, if applicable, for the original study on which the present article is based	17	Funding information and the role of the funders are provided in the Acknowledgements section.

*Give information separately for cases and controls in case-control studies and, if applicable, for exposed and unexposed groups in cohort and cross-sectional studies.
